# Integrated profiling of RUNX3 in intratumoral NK cells activity through bulk and single-cell transcriptomic analysis

**DOI:** 10.3389/fimmu.2026.1787302

**Published:** 2026-04-07

**Authors:** Guofu Lin, Lanlan Lin, Jincan Zhang, Leiyuan Chen, Weitao Hu

**Affiliations:** 1 Department of Pulmonary and Critical Care Medicine, The First Affiliated Hospital of Fujian Medical University, Fuzhou, China; 2 Department of Pulmonary and Critical Care Medicine, National Regional Medical Center, Fujian Medical University, Fuzhou, China; 3 Respiratory Disease Research Institute, The First Affiliated Hospital, Fujian Medical University, Fuzhou, China; 4 The School of Medical Technology and Engineering, Fujian Medical University, Fuzhou, China; 5 Department of Cardiothoracic Surgery, 900th Hospital of PLA Joint Logistic Support Force, Fuzhou, China; 6 Department of Radiation Oncology, Longyan First Affiliated Hospital of Fujian Medical University, Longyan, China; 7 Department of Gastroenterology, The Second Affiliated Hospital of Fujian Medical University, Quanzhou, Fujian, China

**Keywords:** hepatocellular carcinoma, lung adenocarcinoma, NK cell, RUNX3, single-cell RNA sequencing

## Abstract

**Background:**

Natural killer (NK) cells are core components of innate antitumor immunity, the dysfunction of NK cells in the tumor microenvironment is a major obstacle to the antitumor efficacy. Runt-related transcription factor 3 (RUNX3) acts as a critical tumor suppressor and regulates immune cell function, while its biological role in NK cells remains largely unexplored. Herein, we investigated the interaction between RUNX3 and NK cells in tumor microenvironment.

**Methods:**

The Cancer Genome Atlas (TCGA) database was utilized to determine the genetic alteration of RUNX3 in pan-cancer. TIMER and GEPIA website were used to evaluate the correlation between RUNX3 and immune cell infiltration. Single-cell RNA sequencing (scRNA-seq) analysis was applied to characterize RUNX3 expression and pseudotime trajectory in NK cells. *In vitro* experiments were further performed to validate RUNX3’s role in regulating NK cell functions.

**Results:**

RUNX3 was significantly downregulated in lung adenocarcinoma and hepatocellular carcinoma tissues. Clinical analyses have demonstrated that defective RUNX3 expression was correlated with adverse prognosis. Immune infiltration analyses revealed that RUNX3 was positively associated with immune cell infiltration, particularly NK cells and CD8^+^ T cells. scRNA-seq indicated RUNX3 enrichment in intratumoral NK cells, and differential genes of RUNX3 were enriched in the MAPK signaling pathway. Pseudotime trajectory analysis indicated RUNX3 participated in NK cell differentiation. Moreover, RUNX3 overexpression enhanced NK cell viability, chemotactic capacity, cytotoxicity against tumor cells, and secretion of pro-inflammatory cytokines and granzyme B, while upregulating NK cell activation receptors.

**Conclusion:**

Our findings identify RUNX3 as a key regulator of NK cell-mediated antitumor immunity in LUAD and LIHC, providing a novel molecular target for enhancing innate immune surveillance and developing targeted immunotherapies for the aggressive malignancies.

## Introduction

Natural killer (NK) cells, the essential components of innate lymphoid cells, constitute the frontline of innate antitumor immunity and play an irreplaceable role in surveilling and eliminating malignant cells across multiple cancer types (1, 2). Unlike adaptive immune cells, NK cells exert cytotoxicity without prior antigen sensitization, relying on dual recognition of activating and inhibitory surface receptors to distinguish tumor cells from normal cells (3, 4). The antitumor activity of NK cells is primarily mediated by two core mechanisms: direct cell-cell contact-dependent release of cytolytic granules or CD16-initiated ADCC to induce target cell apoptosis (5–7), as well as remodeling the tumor immune microenvironment (TIME) by secretion of pro-inflammatory cytokines like IFN-γ to enhance dendritic cell (DC) maturation and CD8^+^ CTL function and synergize with other immune cells to amplify antitumor immunity (8).

In the context of lung adenocarcinoma (LUAD) and hepatocellular carcinoma (LIHC), two of the most prevalent and lethal malignancies globally with high rates of distant metastasis and poor clinical outcomes (9, 10), NK cell dysfunction is closely linked to tumor progression and treatment resistance. The immunosuppressive TIME impairs NK cell infiltration and effector function, in LIHC, chronic liver inflammation and hepatic stellate cell (HSCs) sustained activation further compromise NK cell-mediated tumor clearance (11, 12). Despite the promising potential of NK cell-based immunotherapies, the limited persistence and functional exhaustion of NK cells in the tumor microenvironment (TME) severely restrict clinical efficacy, highlighting the urgency to identify molecular targets capable of enhancing NK cell antitumor capacity.

The Runt-related transcription factor (RUNX) family, comprising RUNX1, RUNX2, and RUNX3, plays diverse roles in development and immunity (13). Aberrant expression of RUNX3 has long been recognized as a critical tumor suppressor in multiple solid tumors (14). By contrast, RUNX1 mainly contributes to hematopoietic stem cell differentiation (15, 16), and RUNX2 is primarily involved in osteogenesis (17, 18). In tumor cells, RUNX3 regulates cell cycle arrest, stemness, and epithelial-mesenchymal transition (EMT) by modulating downstream molecules such as p21, β-catenin, and E-cadherin (19–21). Promoter methylation-induced downregulation or degradation of RUNX3 is associated with advanced tumor stage and poor prognosis in cancer patients (14, 22, 23). Beyond its cell-intrinsic role in tumor cells, emerging evidence suggested that RUNX3 participated in the regulation of immune cell function, with reports implicating RUNX3 in T cell differentiation and macrophage polarization in the TIME (24–27). However, the biological function of RUNX3 in NK cells remains largely unexplored.

Herein, we initially integrated bioinformatic analyses to profile the expression patterns of RUNX3 across diverse tumor types, while concurrently examining its correlation with tumor-infiltrating immune cell landscapes. Single-cell RNA sequencing (scRNA-seq) was further applied to characterize RUNX3 transcriptomic profiling specifically within NK cells, including pathway enrichment and pseudotime trajectory analyses. Additionally, complementary *in vitro* functional assays validated that RUNX3 was involved in orchestrating the antitumor cytotoxicity exerted by NK cells.

## Materials and methods

### Expression and survival analysis of RUNX3 in pan-cancer

The Cancer Genome Atlas (TCGA, *https://cancergenome.nih.gov*) aggregates sequencing data from a large number of human cancer samples, enabling comprehensive genomic profiling of various cancers (28). RUNX3 mRNA expression was analyzed on 33 cancer types using data sourced from the TCGA databases. The Human Protein Atlas (HPA, *https://www.proteinatlas.org/*) provides comprehensive data on the expression patterns and subcellular localization of human proteins in diverse tissues and cell types (29). The HPA database was utilized to retrieve data regarding RUNX3 mRNA and protein expression in human tissues.

The “survival” package in R was applied to perform Kaplan-Meier survival analysis on the RUNX3^high^ and RUNX3^low^ expression groups across five cancer types, including four essential prognostic endpoints: overall survival (OS), progression-free survival (PFS), post-progression survival (PPS), and recurrence-free survival (RFS).

### Mutation analysis of RUNX3 in pan-cancer

cBioPortal serves as a public online platform dedicated to querying cancer genomics datasets, offering extensive cancer genomics data, encompassing details of gene mutations, copy number variations, and expression differences (30). To characterize the genomic variation profiles of RUNX3 across different cancer types, RUNX3 gene mutation was explored, including the frequency of somatic mutations and their specific mutation sites, aiming to clarify the genomic variation characteristics of RUNX3 in distinct cancer types.

### Protein-protein interaction network analysis

The STRING database acts as an integrated platform for protein-protein interaction (PPI) networks, encompassing both validated and computationally predicted interaction data (31). Equipped with an intuitive interface, STRING enables diverse analyses including PPI network visualization, functional enrichment assays, and genome context-based predictions. We retrieved RUNX3 interaction molecules from the STRING database, and the filtered network data were imported into Cytoscape for visualization. Kyoto Encyclopedia of Genes and Genomes (KEGG) and Gene Ontology (GO) functional enrichment analyses were subsequently employed to visually represent the analysis results via bubble charts.

### Immune infiltration analysis

The TIMER 2.0 database was applied to investigate the relationship between immune cell infiltration and RUNX3 expression. Gene Expression Profiling Interactive Analysis (GEPIA) (*http://gepia.cancer-pku.cn/*) is a visualization website based on TCGA datasets (32). The GEPIA 2021 website was further used for the correlation between RUNX3 and immune cell infiltration. Immune scores and stromal scores were calculated using the ESTIMATE algorithm. The infiltration abundance of immune cells in heterogeneous tumor tissues was assessed using the Microenvironment Cell Population-counter (MCP-counter) algorithm.

### Single-cell sequencing analysis

Raw scRNA-seq count matrices were retrieved from GEO database under GSE140228 (LIHC) and GSE127465 (LUAD), respectively. All analyses were performed using R software (v4.2.1) with the Seurat package (v4.3.0). The filtered count matrices were normalized via the NormalizeData function and scaled using ScaleData. Cell clustering was conducted with FindNeighbors and FindClusters, followed by non-linear dimensionality reduction via RunUMAP to visualize cell populations.

Cell type annotation was achieved by mapping cluster-specific expression of canonical cell-type marker genes. Marker gene expression was visualized via dot plot and violin plot to confirm cluster identities. NK cells were subset from the total cell population, and stratified into RUNX3^high^ and RUNX3^low^ subpopulations based on the median expression of RUNX3. Differentially expressed genes (DEGs) between the two subpopulations were visualized via violin plot.

Pseudotime trajectory analysis of NK cells was performed using the “Slingshot” package (v2.2.0), with UMAP coordinates and Seurat cluster labels as input. The trajectory was initialized using the cluster with the lowest RUNX3 expression as the starting point, and pseudotime values were assigned to each cell. The dynamic expression of RUNX3 along the pseudotime axis was visualized via ggplot, and integrated with trajectory UMAP plots to link RUNX3 dynamics to NK cell differentiation.

### Cell culture and cell infection

The LUAD cell line A549, LIHC cell line HepG2, NK-92MI cell and HEK-293T cells were purchased from Cell Bank of Chinese Academy of Science (Shanghai, China). NK-92MI cells and tumor cells were used as effector cells and target cells, respectively. A549 and HepG2 cells were cultured in RPMI-1640 medium supplemented with 10% FBS, NK-92MI cells were cultured in α-MEM supplemented with 10% FBS, 2 mM L-glutamine, and 100 IU/mL recombinant human interleukin-2 (rhIL-2) to support cell proliferation. Cell culture dishes and conical-bottom centrifuge tubes were purchased from Bioland (China).

For cell infection experiment, RUNX3 short hairpin RNA (sh-RNA) lentivirus, and RUNX3 overexpression lentivirus manufactured by Hanheng Biotechnology (Shanghai, China) were infected into cells in the presence of 8 μg/ml polybrene with 40 multiplicity of infection (MOI). After infection for 16 h, the medium containing virus particles was removed and changed to complete medium. Three days post-infection, GFP expression was observed in three randomly selected fields using a fluorescence microscope. Approximately 90% incubated cells observed GFP staining was considered to be feasible for the following procedure. Optimal concentration of puromycin (Sigma, St. Louis, MO, USA) was confirmed in preliminary experiment and the final concentration was determined as 4 μg/ml. Infection efficiency was guaranteed by RT-qPCR and western blot.

### RNA extraction and RT-qPCR

Total RNA was extracted from cells via TRIzol^®^ Reagent according to the manufacturer’s protocol. Extracted RNA was transcribed into cDNA, and RT-qPCR was performed using the 7500 Real-Time PCR detection system (Applied Biosystems, China). Relative mRNA expression was calculated using 2^-ΔΔCt^ method and normalized to GAPDH expression. RT-qPCR primer sequences were listed as follows: RUNX3 forward: 5’-AGGCAATGACGAGAACTACTCC-3’, reverse: 5’- CGAAGGTCGTTGAACCTGG-3’, GAPDH forward: 5’-GGAGCGAGATCCCTCCAAAAT-3’, reverse: 5’-GGCTGTTGTCATACTTCTCATGG-3’.

### Western blot

Western blot was performed as previously described. Tissue and cell lysates were prepared using RIPA lysis buffer (Beyotime, China). The samples were resolved by SDS-polyacrylamide gel electrophoresis and blotted on PVDF membranes (Millipore, USA). The membranes were blocked with a 5% nonfat milk solution in TBST for 2 h at room temperature. Primary antibodies were incubated at 4°C overnight. The primary antibodies were listed as follows: RUNX3 (1:1000, ab135248, abcam, UK), caspase-3 (1:5000, ab32351, abcam, UK), Bcl-2 (1:2000, ab182858, abcam, UK), Bax (1:1000, bsm-52316R, Bioss, USA), cleaved-caspase3 (1:500, ab32042, abcam, UK), DUSP6 (1:500, ab76310, abcam, UK), p-ERK (1:1000, bs-3330R, Bioss, USA), ERK (1:2000, bsm-61081R, Bioss, USA), β-actin (1:5000, ab8227, abcam, UK). The membranes were incubated with an HRP-conjugated secondary antibody at room temperature for 1 h. The immune complexes were detected using ImageQuant LAS 4000 (GE Healthcare, UK).

### ELISA

Human TNF-α, IFN-γ, and GzmB ELISA kits were purchased from Solarbio Science & Technology Co., Ltd (Beijing, China). Tumor cells were seeded into 24-well plates and incubated overnight, after which NK-92MI cells were added at an effector-to-target (E: T) ratio of 10:1, with all groups set up in triplicate and incubated at 37 °C with 5% CO_2_ for 24 h. Following co-culture, the supernatant of cells was harvested to measure the cytokine secretion according to the manufacturer’s instructions.

### Tumor cell-conditioned medium preparation

Target tumor cells were cultured in DMEM medium containing 10% FBS, 100 U/mL penicillin, and 100 μg/mL streptomycin. When the tumor cells reached 80%-90% confluence, the culture medium was discarded, and the cells were washed twice with PBS. Subsequently, serum-free RPMI 1640 medium was added and the cells were incubated for 24 h. After incubation, the supernatant was collected and filtered through a 0.22 μm sterile filter (Millipore) for sterilization. The conditioned medium was either used immediately or stored at -80°C for no more than 1 month.

### Chemotactic capacity analysis

To assess NK-92MI cell chemotaxis, NK-92MI cells in the logarithmic growth phase were collected by centrifugation, and resuspended in serum-free RPMI 1640 medium to a final concentration of 5×10^5^ cells/mL. Then, 200 μL of the cell suspension was added to the upper compartment of 8-μm Transwell chambers, the lower compartment contained 600μL TCM as chemoattractant. Transwell plates were placed in a humidified incubator at 37°C with 5% CO_2_ for incubation. After 4 h of incubation, migrated cells in the lower compartment were visualized, and the fluorescence intensity was quantified.

### Flow cytometry

HepG2 cell apoptosis was detected via flow cytometry using the Annexin V-FITC/PI apoptosis kit (Beyotime, China). Briefly, HepG2 cells were seeded at density of 1×10^5^ cells/well and resuspended in 195 μL of 1× Annexin V−FITC binding buffer. Subsequently, cells were stained with 5 μl of Annexin V-FITC and 10 μl of PI in the dark at room temperature for 15 min. After staining, 400 μL of 1× binding buffer was added to each tube. Apoptotic cells were analyzed by a flow cytometer (Becton Dickinson, USA).

### Magnetic cell sorting

Primary NK cell sorting procedure was as follows: Peripheral blood mononuclear cells (PBMC) were isolated from patients’ peripheral blood by density gradient cell separation using lymphocyte separation tubes (NEST Biotechnology, Wuxi, China). NK cells were enriched from PBMC by negative magnetic-activated cell sorting using the NK Cell Isolation Kit (Miltenyi Biotec) according to the manufacturer’s protocol. In brief, PBMCs were incubated with a biotin-antibody cocktail targeting non-NK cells, followed by anti-biotin microbeads. The cell suspension was then passed through LS columns (Miltenyi Biotec) placed in a magnetic field, allowing the untouched NK cells to flow through. The purity of the isolated NK cells was assessed by flow cytometry. Cells were stained with anti-CD3-APC (E-AB-F1001E, Elabscience) and anti-CD56-PE (E-AB-F1239D, Elabscience) antibodies and analyzed on a flow cytometer (BD FACSCanto II). NK cells were defined as the CD3^-^CD56^+^ population. The study was approved by the Institutional Ethics. All participants provided informed written consent.

### Co-immunoprecipitation

HEK-293T cells pretreated as indicated were collected and placed into 1.5 mL tubes (NEST Biotechnology, Wuxi, China) with 500 μL Cell Lysis Buffer (Thermo Fisher Scientific) and 10 μg primary antibody of anti-RUNX3 (ab224641, Abcam) or anti-DUSP6 (ab76310, abcam) at 4°C overnight. The cell lysis/antibody mixture was poured into a new 1.5 mL tube containing pre-washed Protein A/G Magnetic Beads (Thermo Fisher Scientific) and incubated at room temperature for 1 h. After overnight incubation, the immunocomplexes were washed twice with PBST (pH =7.4 PBS with 0.1% Triton X-100). Bead-bound proteins were eluted by boiling with 2×SDS loading buffer before being resolved by SDS-PAGE.

### Chromatin immunoprecipitation-qPCR

ChIP-qPCR was performed as described previously. NK-92MI cells were fixed in 1.5% formaldehyde for 15 min at room temperature and quenched with 125 mM glycine. After cell lysis, the chromatin was fragmented into 100–500 bp by Bioruptor Sonicator (Diagenode), and protein-DNA complexes were immunoprecipitated by 5 μg RUNX3 anti-body or 2 μg anti-IgG antibody conjugated with Dynabeads Protein G (Invitrogen) on a rotator at 4°C overnight. After washing, reversal of crosslink and DNA purification, equal amounts of IP (by RUNX3 antibody or IgG control) and input DNA were used as templates for conventional PCR assay using specific primers targeting a region within 100 bp of the putative binding site.

### Dual luciferase reporter assay

The promoter region of DUSP6 was amplified by PCR and cloned into pGL3 basic vector (Promega, Madison, USA). HEK-293T cells were transfected with pGL3-DUSP6-promoter, pRL-TK, pGL3-Basic, pcDNA3.1-TLE1, and pcDNA3.1-RUNX3 using Lipofectamine 3000 transfection reagent (Invitrogen). Dual luciferase reporter assay was performed using Dual Luciferase Reporter Assay System (Promega). Renilla luciferase activity was used to normalize transfection efficiency.

### Statistical analysis

Statistical analysis was performed using SPSS 21.0 statistical software. All quantitative data are expressed as mean ± SD from at least three independent experiments. The t-test was performed for comparisons between two groups and one-way analysis of variance (ANOVA) followed by Tukey’s *post hoc* test was used for comparisons among multiple groups. The *P* value less than 0.05 was considered statistically significant.

## Results

### Expression profile and diagnostic value of RUNX3 in pan-cancer

**Figure 1:**
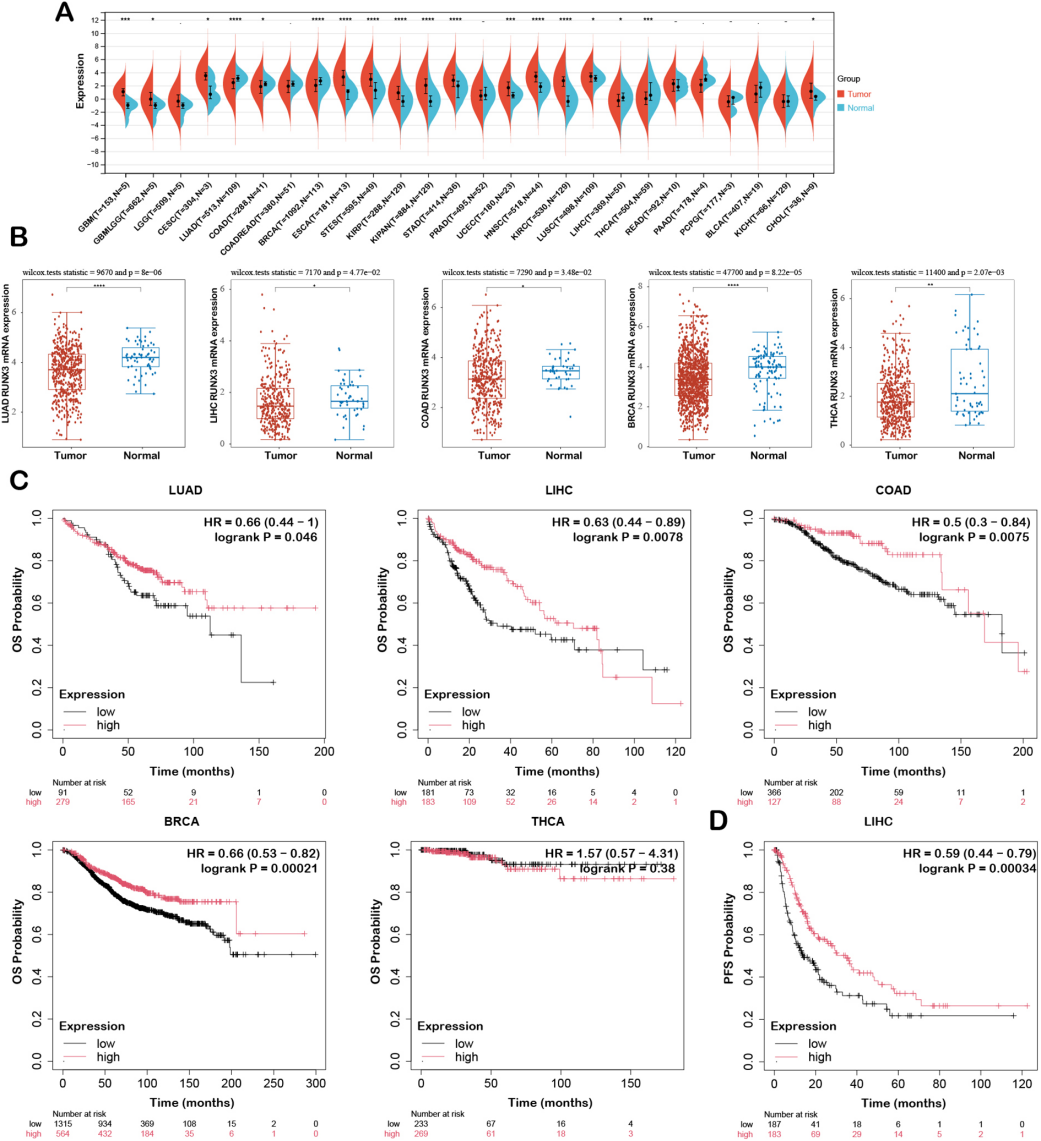
Pan-cancer expression profile of RUNX3 and the association with patient prognosis. **(A)** Violin plot displaying RUNX3 mRNA expression levels across diverse cancer types. **(B)** Differential RUNX3 mRNA expression between tumor and adjacent normal tissues in representative cancers. **(C)** Kaplan-Meier curves for overall survival (OS) of patients with LUAD, LIHC, COAD, BRCA, and THCA. **(D)** Kaplan-Meier curve for progression-free survival (PFS) of LIHC patients. *P < 0.05, **P < 0.01, ***P < 0.001, ****P < 0.0001.

To explore the expression pattern of RUNX3 in pan-cancer, we analyzed the transcriptomic data from TCGA database. The results indicated that RUNX3 mRNA expression was predominantly decreased in lung adenocarcinoma (LUAD), liver hepatocellular carcinoma (LIHC), colon adenocarcinoma (COAD), breast invasive carcinoma (BRCA), and thyroid carcinoma (THCA) compared with the corresponding normal tissues, while the expression was also elevated in multiple other cancers (**Figures 1A, B**). Given that RUNX3 is recognized as a tumor suppressor gene in the tumor microenvironment, we intended to investigate how RUNX3 modulates tumor progression. Therefore, we further performed analysis on tumors with RUNX3 low expression for subsequent investigation.

Analysis of the HPA database revealed that RUNX3 was expressed in diverse organs and tissues of the human body. Specifically, RUNX3 mRNA was mainly detected in bone marrow, lymph node, spleen, tonsil, appendix, small intestine, thymus, and skin, most of which are immune-related organs (**Supplementary Figures S1A, B**). In contrast, RUNX3 protein was primarily localized in small intestine, tonsil, gallbladder, appendix, spleen, and lymph node (**Supplementary Figure S1C**). Immunohistochemical images from the HPA database were also utilized to assess the protein expression of RUNX3 (**Supplementary Figure S2A**). As DNA methylation is an essential epigenetic regulation mechanism of gene expression, we assessed the promoter methylation level of RUNX3 and found that methylation levels of the RUNX3 promoter in tumors were significantly upregulated compared with normal tissues (**Supplementary Figure S2B**).

To investigate the clinical relevance of RUNX3 expression in tumors, we further analyzed its expression patterns across different clinical stages. The results demonstrated that RUNX3 expression was associated with tumor recurrence in LUAD, LIHC, and BRCA, and with metastasis in LUAD and THCA, highlighting its potential regulatory role in tumor progression (**Supplementary Figure S3**). The Kaplan-Meier plot analysis was further applied to predict the prognostic value of RUNX3 in cancer patients. It was revealed that the higher level of RUNX3 was significantly associated with better OS (**Figure 1C**). Consistently, Kaplan-Meier plotter data also supported that RUNX3 was a potential favorable prognostic biomarker for PFS (HR = 0.59, log-rank *P* = 0.00034) in LIHC (**Figure 1D**). Specifically, the high-expression of RUNX3 exhibited better RFS in LIHC, COAD, BRCA, whereas no significant correlation was observed with PPS (**Supplementary Figures S4A, B**). Given the significant prognostic impact of RUNX3 in LIHC and LUAD, we subsequently conducted more in-depth investigations on the two malignancies.

### Mutation analysis of RUNX3 in pan-cancer

**Figure 2:**
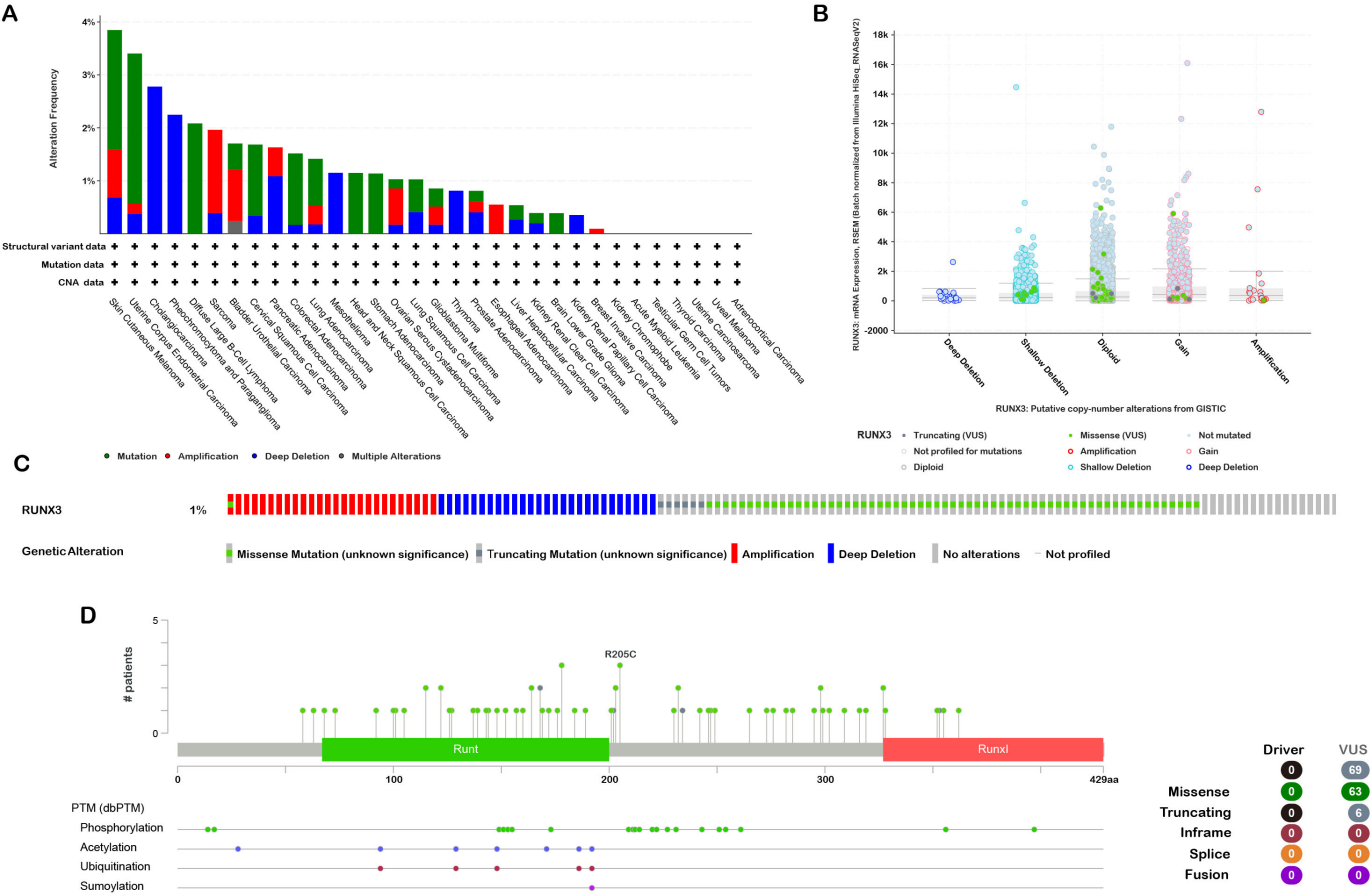
Mutation assessment for RUNX3 using the cBioPortal tool. **(A)** RUNX3 mutation types and frequency. **(B)** RUNX3 copy number alterations (CNA) types. **(C)** OncoPrint visual summary of RUNX3 structural variant, mutations, and copy-number alterations. **(D)** The mutation number and sites of the RUNX3 genetic alterations.

To investigate the genetic alterations of RUNX3 across various cancers, we analyzed data from the cBioPortal platform (**Figure 2**). As shown in **Figure 2A**, skin cutaneous melanoma (SKCM) and uterine corpus endometrial carcinoma (UCEC) exhibited relatively high mutation frequencies of RUNX3, with deep deletions and amplifications being the prominent alteration types in different cancer types.

For putative copy number alteration (CNA) of RUNX3, the most common patterns included deep deletion, shallow deletion, diploidy, gain, and amplification (**Figure 2B**). Regarding the distribution of genetic alterations along the RUNX3, deep deletions and amplifications were the predominant alteration types in multiple cancer types, and mutations were also observed in specific regions (**Figure 2C**). Subsequently, we explored the mutation sites, types, and frequencies of RUNX3. A total of 69 mutation sites were identified, with missense mutations being the most frequent (63 samples), followed by truncating mutations. The R205C mutation was among the notable missense mutation sites. Additionally, RUNX3 was found to be regulated by post-translational modifications, including phosphorylation, acetylation, ubiquitination, and sumoylation (**Figure 2D**), which may reveal the molecular mechanism of RUNX3 in cancer development.

### Function enrichment analysis of RUNX3

**Figure 3:**
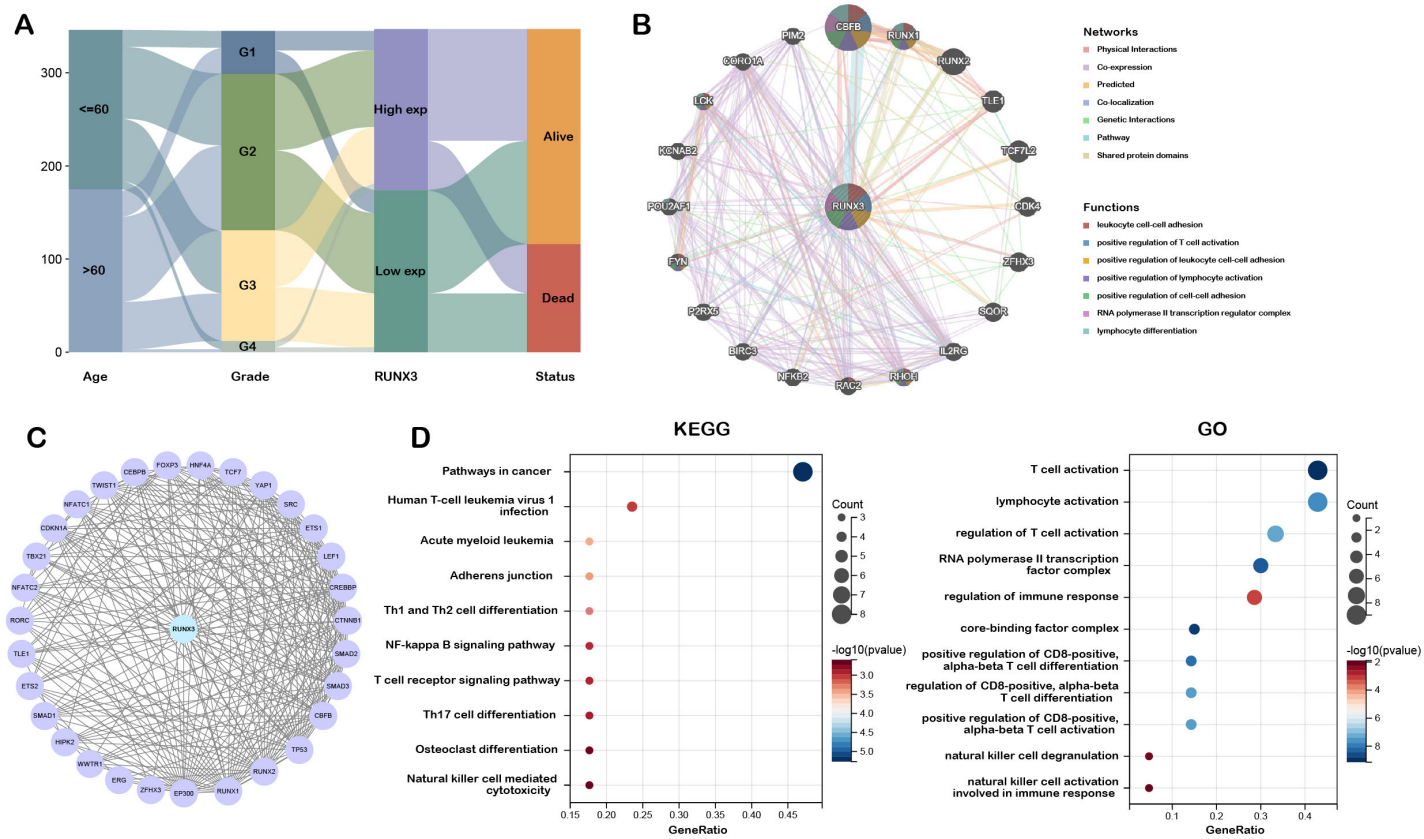
PPI network and functional annotation of RUNX3. **(A)** Sankey diagram visualization of relationships between RUNX3 expression and clinical characteristics of LIHC patients. **(B)** RUNX3 co-expression network constructed by GeneMANIA. **(C)** The PPI network of RUNX3 generated by Cytoscape. **(D)** GO and KEGG enrichment analysis of RUNX3 co-expressed molecules.

A Sankey diagram was subsequently constructed to characterize the association between the clinical relevance and functional landscape of RUNX3. As shown in **Figure 3A**, high expression of RUNX3 was potentially associated with early tumor stage and favorable survival outcomes. To further elucidate the biological implications of RUNX3, we utilized GeneMANIA to generate the protein-protein interaction network. The result indicated that RUNX3 was primarily co-expressed with CBFB, RUNX1, RUNX2, and TLE1. Functional prediction revealed that these proteins were all involved in the leukocyte cell-cell adhesion, positive regulation of T cell activation, and lymphocyte differentiation, underscoring the pleiotropic roles of RUNX3 in immune processes (**Figure 3B**). Genes potentially interacting with RUNX3 were further retrieved from the STRING database (**Figure 3C**), and KEGG pathway and GO enrichment analyses on these RUNX3-interacting genes were conducted. KEGG analysis revealed that RUNX3 correlated with pathways in cancer, T cell differentiation, T cell receptor signaling pathway, and NK cell mediated cytotoxicity. Concordantly, GO annotation indicated that RUNX3 was related to T cell activation, lymphocyte activation, regulation of immune response, and natural killer cell activation (**Figure 3D**). Collectively, the aforementioned consequences suggested the potential mechanisms underlying RUNX3’s role in tumorigenesis and immune modulation.

### RUNX3 mediated immune infiltration of LIHC and LUAD

**Figure 4:**
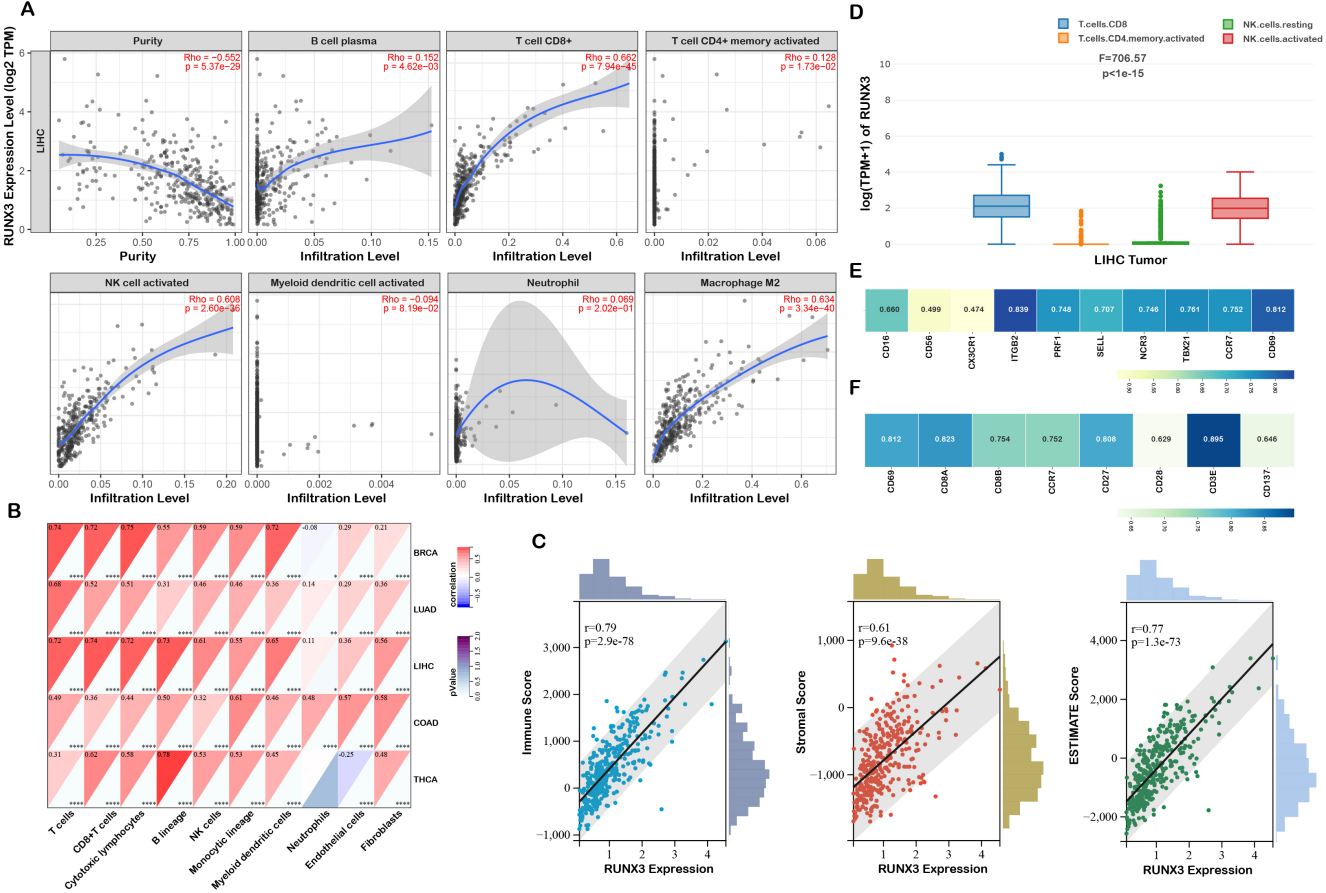
Characteristics of RUNX3 in immune cells infiltration. **(A)** Relationship between RUNX3 expression and immune infiltration level generated from TIMER. **(B)** Clustering of MCP-counter scores for the correlation of RUNX3 with immune and non-immune stromal cell populations. **(C)** Differential expression level of RUNX3 with immune scores and stromal scores. **(D)** RUNX3 expression levels in different immune cells. **(E, F)** Correlations between RUNX3 expression and cell surface markers of NK and CD8^+^ T cells.

The TIMER database was applied to preliminarily explore whether RUNX3 was involved in immune infiltration. The results revealed that RUNX3 was positively correlated with immune cell infiltration in both LIHC and LUAD, particularly in T cells and activated NK cells (**Figure 4A**; **Supplementary Figure S5A**). We further utilized the MCP counter approach to quantify the infiltration levels of immune cells with RUNX3 expression. MCP counter is a transcriptome-based computational method that quantifies the abundance of eight major immune and two stromal cell populations in RNA-seq data from tumor bulk samples (33). As shown in **Figure 4B**, RUNX3 was observed to be differentially expressed in immune cells. Subsequently, the ESTIMATE algorithm was utilized to estimate infiltrating immune cells and stromal cells in the tumor microenvironment of LIHC and LUAD. The results demonstrated that RUNX3 expression was positively correlated with the immune score and stromal score (**Figure 4C**; **Supplementary Figure S5B**), indicating that RUNX3 participated in maintaining TME homeostasis. We further explored the correlations between RUNX3 and immune cells markers via GEPIA database. The result illustrated that RUNX3 was enriched in CD8^+^T cells and activated NK cells (**Figure 4D**; **Supplementary Figure S5C**). Collectively, these findings confirmed that RUNX3 is an immune-related molecule closely associated with T cells and NK cells. We then further investigated the cell surface markers altered with RUNX3 activation, and found that RUNX3 was positively correlated with NK cell and CD8^+^T cell markers (**Figures 4E, F**; **Supplementary Figures S5D, E**).

### Single-cell expression distribution and cluster enrichment analysis of RUNX3

**Figure 5:**
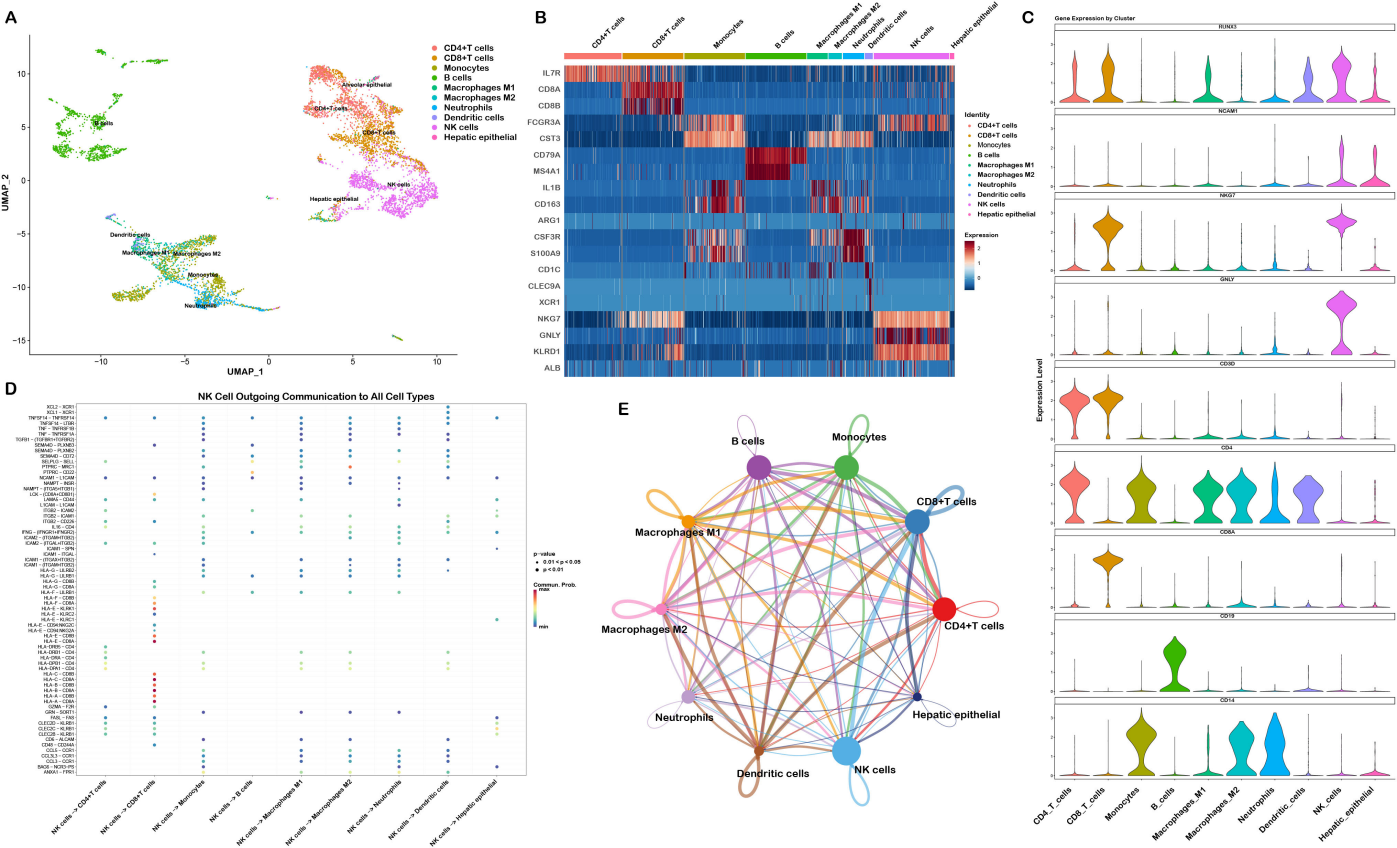
Single-cell analysis of RUNX3 expression pattern and intercellular communication in NK cells of LIHC. **(A)** UMAP plot of the LIHC scRNA-seq dataset GSE140228. **(B)** Heatmap of cell type-specific marker gene expression across distinct cell populations. **(C)** Violin plots showing the expression profiles of RUNX3 and specific marker genes across all annotated cell types. **(D)** Heatmap of outgoing intercellular communication signals originating from NK cells. **(E)** Cell-cell communication network diagram illustrating interactions between NK cells and other tumor-infiltrating cell populations.

To identify the molecular features of RUNX3 in LIHC and LUAD at single-cell level, we analyzed the single-cell RNA-seq data from GSE140228 and GSE127465. We identified multiple transcriptionally distinct cell populations of hepatocellular carcinoma, including CD4+T cells, CD8^+^T cells, B cells, monocytes, macrophages, neutrophils, dendritic cells, NK cells, and hepatic epithelial cells using dimensionality reduction (**Figure 5A**). The identity of each cell cluster was confirmed based on the transcriptional expression of canonical cell-type marker genes (**Figure 5B**; **Supplementary Figures S6A, B**). Furthermore, we profiled the expression distribution of RUNX3 and cluster-specific signature genes and found that RUNX3 was predominantly enriched in CD8^+^T cells and NK cells, particularly in NK cells, indicating its potential functional implications in NK cell biology (**Figure 5C**), while signature genes exhibited strict cluster-specific expression patterns, consistent with the aforementioned marker gene validation (**Supplementary Figures S6C, D**). To characterize the outgoing intercellular communication networks of NK cells, we analyzed the ligand-receptor interaction profiles. The heatmap depicted the communication probability of NK cells interacting with various cell populations. Notably, the crosstalk between NK cells and CD8^+^T cells exhibited the highest communication probability (**Figure 5D**). Additionally, the intercellular interaction network revealed widespread signaling crosstalk among all identified clusters, with CD8^+^T cells and NK cells serving as central hubs (**Figure 5E**).

**Figure 6:**
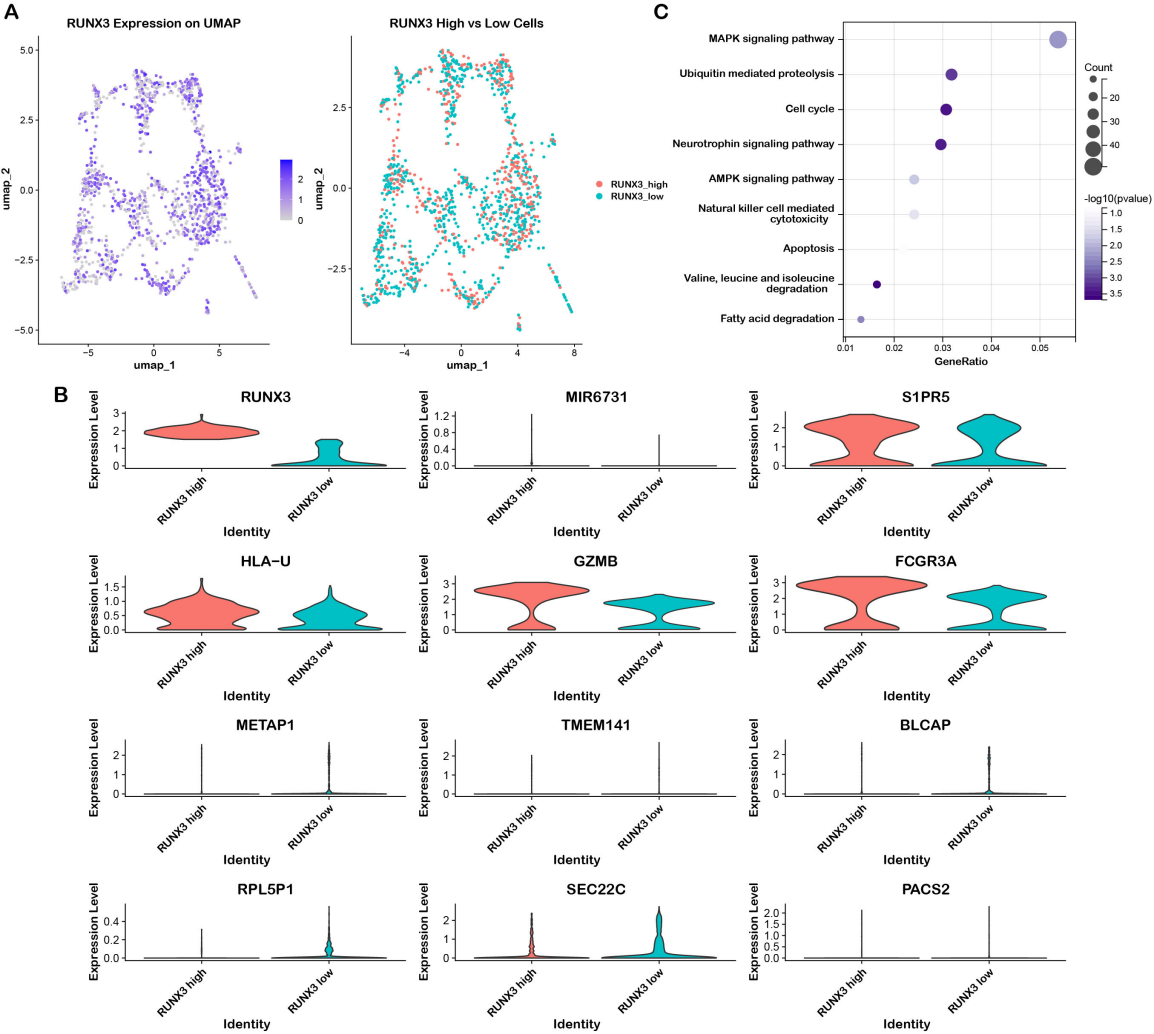
Single-cell analysis of RUNX3 associated differential gene profiles and functional pathway enrichment. **(A)** UMAP plots illustrating RUNX3 expression and cell subset stratification. **(B)** Violin plots displaying the expression levels of RUNX3 and its associated differential genes. **(C)** Functional pathway enrichment analysis for genes associated with RUNX3 expression.

To investigate the functional mechanisms of RUNX3 in NK cells, we stratified NK cells into two subsets based on RUNX3 expression levels. As shown in **Figure 6A**, UMAP visualization clearly illustrated the distribution pattern of RUNX3 in NK cells and distinguished the RUNX3-high and RUNX3-low subpopulations. Subsequently, we performed differential gene analysis on these subsets. The results showed that multiple genes including MIR6731, S1PR5, HLA-U, GZMB, and FCGR3A exhibited distinct expression differences, suggesting that RUNX3 may be involved in regulating the expression of molecules associated with NK cell cytotoxicity (**Figure 6B**). We further conducted pathway enrichment analysis on the differentially expressed genes. The results indicated that the differential genes were significantly enriched in the MAPK signaling pathway (**Figure 6C**), implying that RUNX3 may influence the functional phenotypes of NK cells by regulating the MAPK signaling pathway.

**Figure 7:**
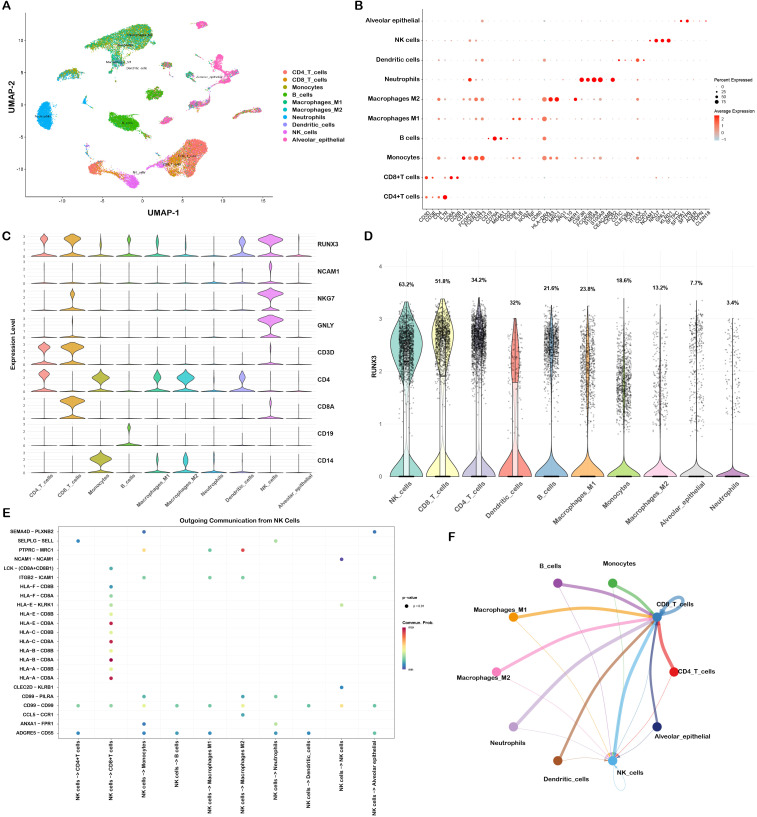
Single-cell analysis of RUNX3 expression pattern and intercellular communication in NK cells of LUAD. **(A)** UMAP plot of the LUAD scRNA-seq dataset GSE127465. **(B)** Dot plot showing marker genes of immune cell subsets in the LUAD single-cell dataset. **(C)** Violin plots showing the expression profiles of RUNX3 and specific marker genes across all annotated cell types. **(D)** Swarm plot showing the distribution of RUNX3 expression in annotated cell types. **(E)** Heatmap of outgoing intercellular communication signals originating from NK cells. **(F)** Cell-cell communication network diagram illustrating interactions between NK cells and other tumor-infiltrating cell populations.

We subsequently conducted scRNA-seq analysis based on RUNX3 in a LUAD dataset. We identified multiple distinct cell populations in the LUAD microenvironment via UMAP dimensionality reduction (**Figure 7A**). The expression pattern of cell-type-specific marker genes validated the identity of each cluster (**Figure 7B**; **Supplementary Figure S7**). Violin plots further illustrated the expression distribution of RUNX3 and canonical signature genes across clusters, indicating the specific expression pattern of RUNX3, particularly in NK cells (**Figure 7C**). Quantification of RUNX3 expression across all clusters also revealed that NK cells harbored the highest proportion of RUNX3-high cells (**Figure 7D**). Moreover, analysis of outgoing communication from NK cells (**Figure 7E**) and the intercellular interaction network (**Figure 7F**) demonstrated extensive crosstalk between NK cells and CD8^+^T cells, implying that RUNX3 may shape LUAD immune microenvironments by regulating NK cell-mediated intercellular communication. To further characterize the RUNX3-dependent intercellular communication between NK cells and CD8^+^ T cells, we analyzed the differential ligand expression in NK subsets and their corresponding receptor-ligand interactions. Violin plots showed that ligands such as SELPLG, HLA-F, CD99, and HLA class I molecules (HLA-A, HLA-B, HLA-E) were upregulated in RUNX3-high NK cells, while CLEC2D and LCK were enriched in RUNX3-low NK cells (**Supplementary Figure S8A**). Cell-cell communication analysis further revealed that the COL6A2–CD44 and COL6A2–ITGA1/ITGB1 pairs exhibited significantly stronger interaction probabilities in RUNX3-low NK cells compared to RUNX3-high NK cells (**Supplementary Figure S8B**). Meanwhile, HLA class I-receptor axes displayed robust crosstalk in both subsets, highlighting RUNX3 as a key regulator of NK-CD8^+^ T cell communication via extracellular matrix and antigen recognition pathways. We further performed functional enrichment analyses based on the DEGs between RUNX3-high and RUNX3-low groups, and the result suggested that these DEGs were mainly enriched in MAPK signaling pathway (**Supplementary Figures S9A, B**), which was consistent with the enrichment results in LIHC.

### Single-cell pseudotime trajectory analysis of NK cells by Slingshot

**Figure 8:**
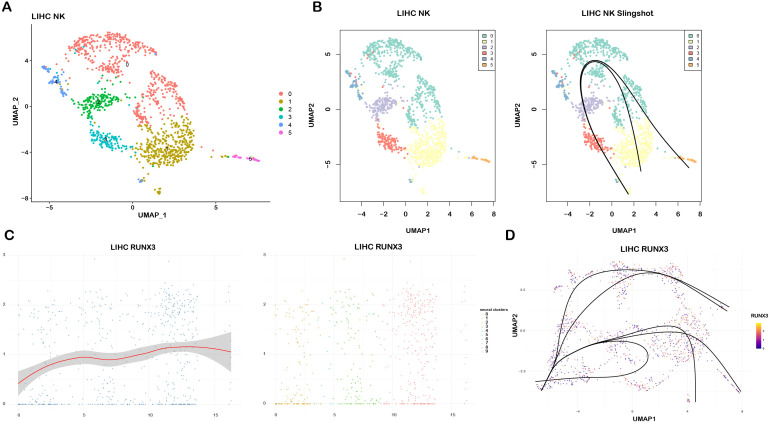
Pseudotime trajectory analysis of RUNX3-associated NK cell differentiation in LIHC GSE140228 dataset. **(A)** UMAP plot illustrating transcriptionally distinct NK cell subpopulations in LIHC microenvironment. **(B)** Slingshot-inferred pseudotime trajectories depicting the developmental differentiation paths of NK cells. **(C)** Dynamic expression profile of RUNX3 along the pseudotime axis and the spatial distribution of RUNX3 expression across NK cells. **(D)** Integrated pseudotime trajectory plot revealing the coupling between RUNX3 expression dynamics and the differentiation trajectories of NK cells.

To elucidate the RUNX3-mediated differentiation dynamics of NK cells in LIHC and LUAD, we performed pseudotime trajectory analysis. As depicted in **Figure 8A** and **Supplementary Figures S10A**, UMAP visualization revealed transcriptionally distinct NK cell subpopulations, each annotated by unique color codes. Subsequently, pseudotime trajectory inference using Slingshot delineated the developmental trajectories of these NK cell subpopulations (**Figure 8B**; **Supplementary Figure S10B**). To dissect the regulatory role of RUNX3 in NK cell differentiation, we profiled its expression across the pseudotime axis and visualized its correlation with the differentiation trajectories (**Figures 8C, D**). The results demonstrated that RUNX3 expression displayed dynamic alterations along the pseudotime progression, and its expression pattern was tightly coupled with the differentiation trajectories of NK cells (**Supplementary Figures S10C, D**). Collectively, these findings collectively imply that RUNX3 may play a regulatory role in the NK cell differentiation within the tumor microenvironment.

### Verification of RUNX3 in modulating the phenotypes of NK cells *in vitro*

**Figure 9:**
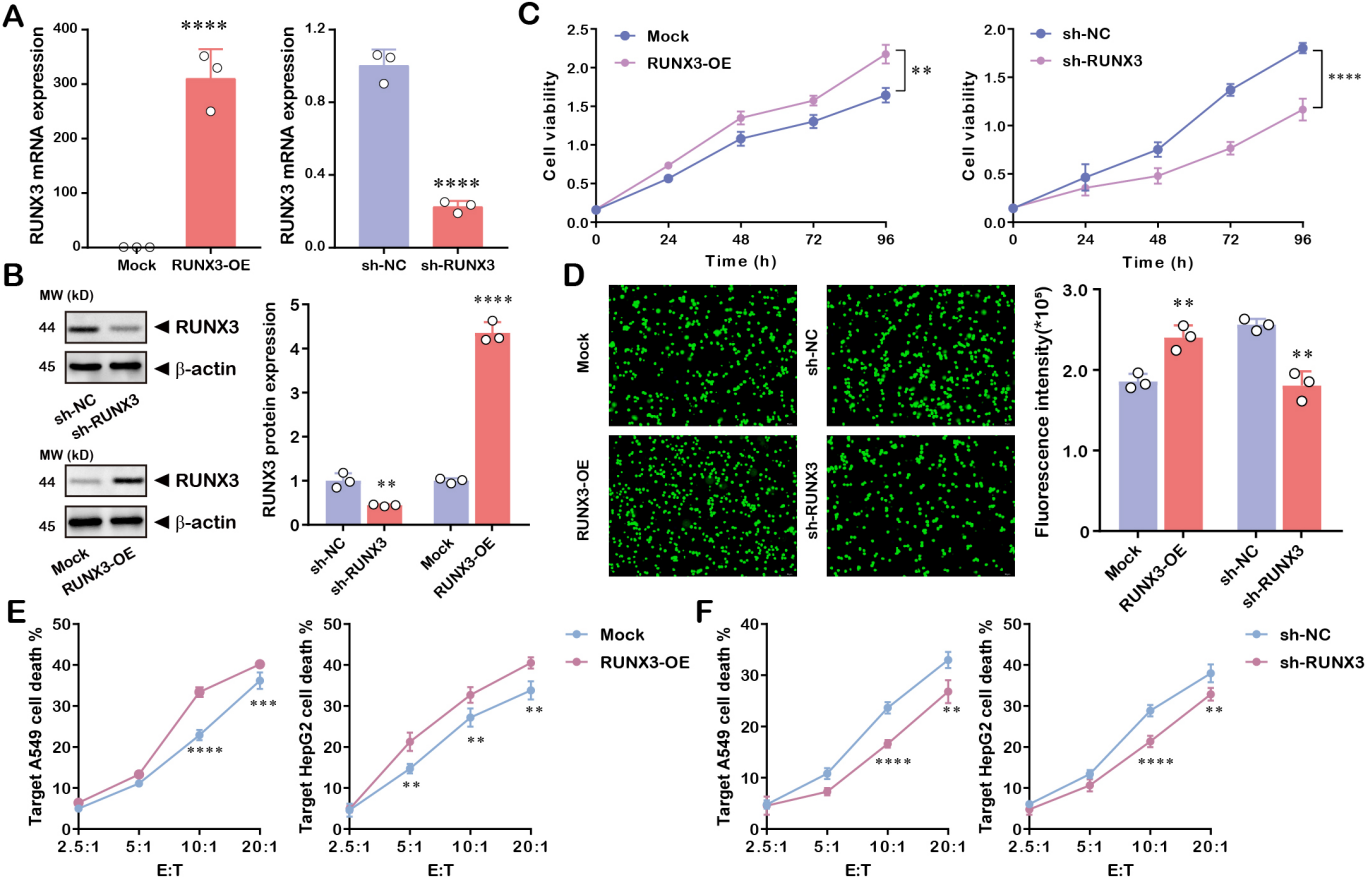
RUNX3 regulates viability and cytotoxicity of NK cells. **(A, B)** RT-qPCR and Western blot of RUNX3 expression in RUNX3-overexpressing or RUNX3-knockdown NK-92MI cells. **(C)** CCK-8 assay assessing the viability of NK-92MI cells over 96 h. **(D)** Chemotaxis assay evaluating the chemotactic capacity of NK-92MI cells. **(E, F)** Cytotoxicity assay measuring the killing activity of RUNX3 overexpressed or downregulated NK-92MI cells against tumor cells across different effector-to-target (E: T) ratios. **P < 0.01, ***P < 0.001, ****P < 0.0001.

To validate the biological function of RUNX3 in NK cells, we initially established RUNX3-overexpressing (RUNX3-OE) and RUNX3-knockdown (sh-RUNX3) NK cell models. The efficiency of genetic manipulation was confirmed by RT-qPCR and Western blot (**Figures 9A, B**). We subsequently assessed NK cell viability. The results indicated that cell viability was significantly increased after RUNX3 overexpression. Conversely, RUNX3 knockdown contributed to a notable reduction in NK cell viability (**Figure 9C**), indicating that RUNX3 positively regulates NK cell viability. The chemotactic capacity of NK cells was evaluated using fluorescence-based chemotaxis assays. Fluorescence imaging revealed altered chemotactic recruitment of NK cells upon RUNX3 modulation (**Figure 9D**). The cytotoxic activity of NK cells against HepG2 cells at varying E: T ratios was further detected. The results demonstrated that killing rates against target cells increased with escalating E:T ratios, and were significantly higher than those of controls (**Figure 9E**). In contrast, RUNX3 knockdown in NK cells resulted in a marked reduction in cytotoxicity (**Figure 9F**), confirming that RUNX3 enhances the cytotoxic function of NK cells.

**Figure 10:**
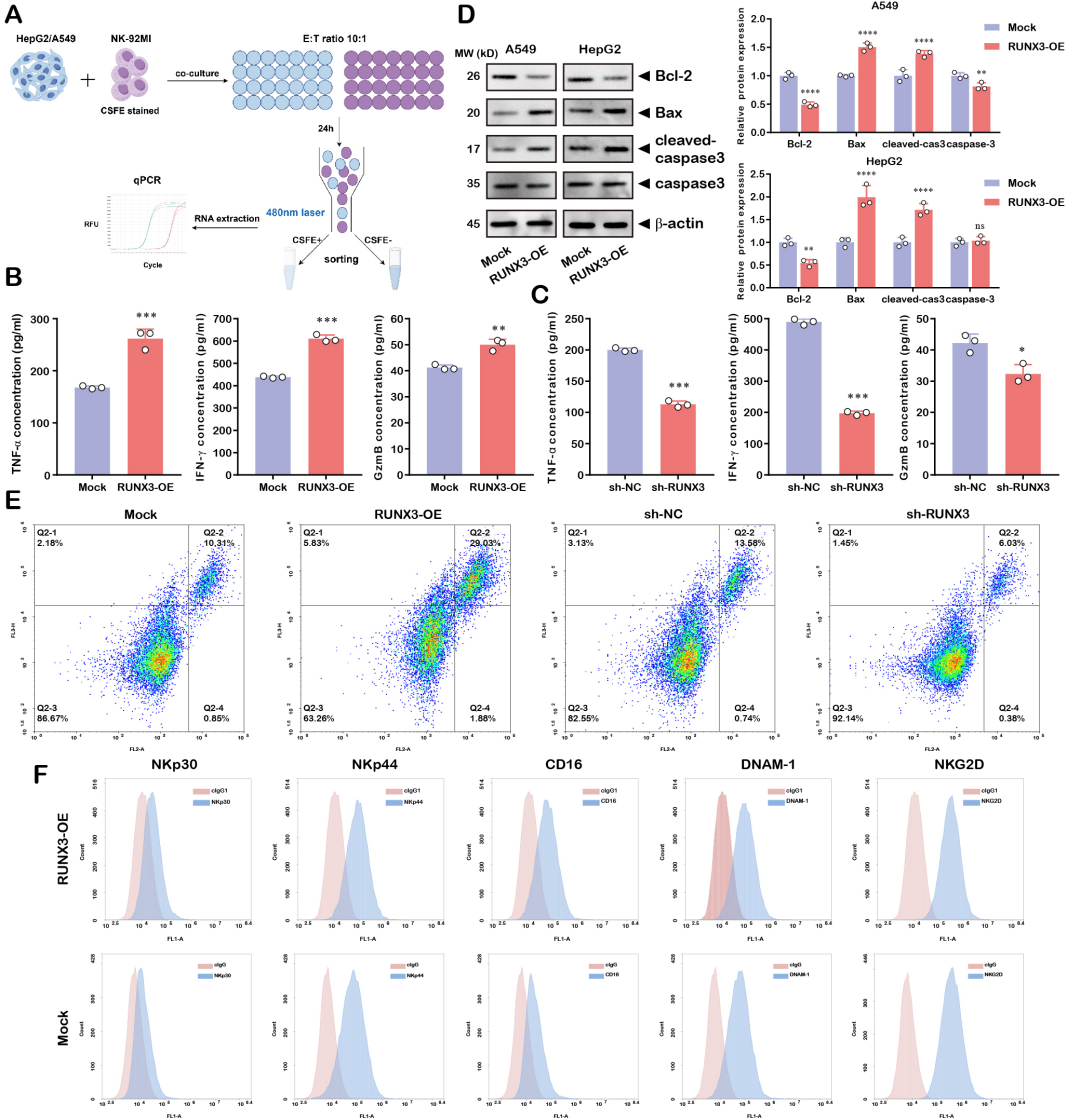
RUNX3 modulates cytokine secretion, target cell apoptosis induction and activation receptor expression in NK-92MI cells. **(A)** Schematic of CFSE-labeled NK-92MI co-cultured with HepG2/A549 tumor cells. **(B, C)** Quantification of cytokine secretion in NK-92MI co-culture supernatants. **(D)** Apoptosis-related protein expression in target tumor cells post co-culture. **(E)** Flow cytometric apoptosis analysis of tumor cells co-cultured with NK cells. **(F)** Flow cytometry analysis of NK cell activation receptor expression. *P < 0.05, **P < 0.01, ***P < 0.001, ****P < 0.0001.

Subsequently, we co-cultured CFSE-labeled NK-92MI cells with HepG2 and A549 cells at an E:T ratio of 10:1 for 24 hours (**Figure 10A**). We quantified the secretion of NK cell effector molecules in co-culture supernatants. The concentrations of pro-inflammatory cytokines (TNF-α, IFN-γ) and the cytolytic granule protein GzmB were significantly elevated in RUNX3-overexpressing NK cells, while RUNX3 knockdown led to opposite results (**Figures 10B, C**). To assess NK cell-induced target cell apoptosis, we analyzed apoptotic regulatory proteins in tumor cells after co-culture. Western blot showed that RUNX3 overexpression promoted target cell apoptosis, while RUNX3 knockdown exerted an inhibitory effect on cell apoptosis (**Figures 10D, E**). NK cell effector activity is critically dependent on the expression of the surface receptors, which primarily mediate target cell recognition and the transmission of activating signals. Therefore, we evaluated the expression of critical NK cell surface activation receptors via flow cytometry. The result indicated that RUNX3 overexpression significantly upregulated the surface levels of the receptors (**Figure 10F**).

Considering the NK-92MI cell line may not fully recapitulate the phenotypic characteristics of primary NK cells, we further isolated NK cells from peripheral blood to validate the essential results (**Supplementary Figure S11A**). We established RUNX3-overexpressing primary NK cell models via lentiviral transduction. Western blot analysis revealed RUNX3 significantly increased expression of apoptotic molecules compared to controls (**Supplementary Figure S11B**). These primary NK cells presented higher cytotoxic activity against tumor cells across varying E: T ratios (**Supplementary Figure S11C**). Additionally, ELISA assays demonstrated that the secretion of key effector cytokines, particularly IFN-γ and TNF-α, was markedly elevated in the co-culture supernatants of RUNX3-overexpressing primary NK cells (**Supplementary Figure S11D**).

### RUNX3 regulated NK cell cytotoxicity via the MAPK pathway

**Figure 11:**
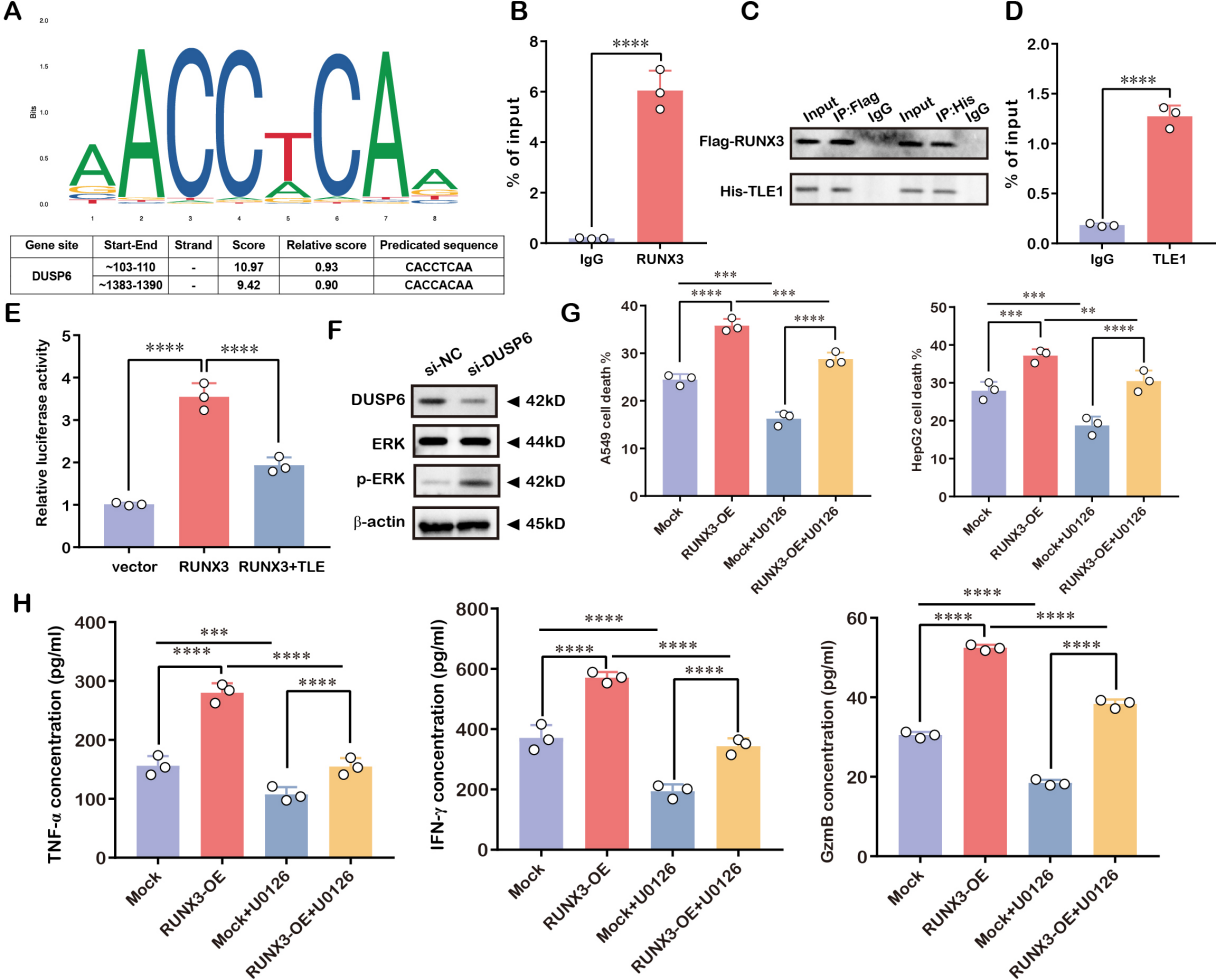
RUNX3/TLE1 complex repressed DUSP6 to activate MAPK signaling and promote NK cell cytotoxicity. **(A)** JASPAR prediction of RUNX3-binding sites in the DUSP6 promoter. **(B)** ChIP-PCR validation of RUNX3-DUSP6 promoter binding. **(C)** Co-IP confirmation of RUNX3-TLE1 interaction. **(D)** ChIP-PCR validation of TLE1-DUSP6 promoter binding. **(E)** Dual-luciferase assay showing synergistic repression of DUSP6 by RUNX3 and TLE1. **(F)** Western blot showing p-ERK expression after DUSP6 knockdown. **(G)** NK cell cytotoxicity assay against A549 and HepG2 targets with U0126. **(H)** ELISA measurement of TNF-α, IFN-γ, and GzmB secretion by NK cells. **P < 0.01, ***P < 0.001, ****P < 0.0001.

To elucidate the regulatory mechanism of RUNX3 in NK cell-mediated tumor immunity, we first performed *in silico* prediction of RUNX3 binding sites using the JASPAR database. As shown in **Figure 11A**, RUNX3-binding motifs were identified within the promoter region of DUSP6, a negative regulator of the MAPK pathway. ChIP-qPCR assays confirmed the direct physical association of RUNX3 with the DUSP6 promoter (**Figure 11B**). Given our prior PPI network that identified TLE1 as a putative negative regulator of RUNX3, we hypothesized that RUNX3 might collaborate with TLE1 to transcriptionally repress DUSP6. Co-IP assays validated the physical interaction between RUNX3 and TLE1 (**Figure 11C**). Subsequent ChIP-qPCR demonstrated that TLE1 also bound to the DUSP6 promoter (**Figure 11D**), and dual-luciferase reporter assays revealed that TLE1 synergized with RUNX3 to suppress DUSP6 transcriptional activity (**Figure 11E**).

To explore the functional consequence of DUSP6 repression, we knocked down DUSP6 expression and observed a marked increase in p-ERK levels, indicating activation of the MAPK pathway (**Figure 11F**). In addition, we further pretreated NK cells with the MAPK pathway inhibitor U0126 for 24h before conducting cytotoxicity assays. The results indicated that pharmacological inhibition of MAPK signaling with 10 μM U0126 significantly impaired the cytotoxic capacity of NK cells against target cells (**Figure 11G**), as well as the secretion of pro-inflammatory cytokines and cytotoxic granzyme B (**Figure 11H**). Collectively, these findings demonstrated that RUNX3 cooperated with TLE1 to transcriptionally inhibit DUSP6, thereby activating the MAPK signaling cascade to promote the cytotoxic effector function of NK cells in the tumor microenvironment.

## Discussion

As essential components of innate immune surveillance, NK cells exert profound control over tumor metastasis by modulating the immunomodulatory landscape of TIME (34, 35). Existing studies have sporadically documented RUNX3 expression in NK cell subsets (36, 37), while the impact on NK cell cytotoxicity, and cytokine secretion has not been systematically elucidated. Herein, we identified that RUNX3 expression was markedly downregulated in both LUAD and LIHC tissues. Notably, scRNA-seq revealed that RUNX3 was enriched in intratumoral NK cells. Pathway correlation analyses further indicated that RUNX3 exhibited a positive association with the MAPK signaling pathway. Subsequently, complementary *in vitro* assays confirmed that RUNX3 was involved in modulating the secretion of pro-inflammatory cytokines by NK cells and enhances the cytotoxic activity against tumor cells.

Accumulating evidence has revealed that the genetic expression of RUNX3 is highly context-dependent, exhibiting marked heterogeneity across different cancer types. Studies have demonstrated that upregulation of RUNX3 expression inhibited the proliferation, migration, and invasion of cervical cancer cells, whereas knockdown of RUNX3 promotes these malignant behaviors (38). Mechanistically, RUNX3 exerted its tumor-suppressive effects via various regulatory axes, including the LINC00657/miR-20a-5p/RUNX3 pathway (39). On the other hand, it has been demonstrated that RUNX3 was downregulated in hepatocellular carcinoma (HCC), and low expression of RUNX3 was directly associated with the EMT process (20). In colorectal cancer (CRC), RUNX3 was co-downregulated with circ-METTL3 and PER3, and the coordinated downregulation promoted CRC cell proliferation and metastasis by disrupting a tumor-suppressive regulatory network (40). In addition, RUNX3 downregulation has also been implicated in gastric cancer progression, where the deletion mutations R122C drive pre-cancerous lesions by disrupting epithelial stem cell homeostasis (41). Consistent with previous characterization of RUNX3 as a tumor suppressor, our findings confirmed that RUNX3 was downregulated in LIHC and LUAD, with its low expression significantly associated with unfavorable prognosis. Epigenetically, RUNX3 promoter methylation-induced silencing emerged as a prevalent oncogenic event in multiple malignancies, correlating with reduced CD8^+^ T cell infiltration and resistance to anti-PD-1 immunotherapy (24), highlighting the potential of RUNX3 as a predictive biomarker beyond its prognostic value.

NK cells and CD8^+^ T cells are core components of the anti-tumor immune response, and the functional activation and intercellular crosstalk are crucial for effective tumor immune surveillance (42, 43). Herein, we identified that RUNX3 was enriched in NK cells, and was closely associated with cytotoxic activity. Emerging evidence has increasingly linked RUNX3 to immune regulation. For instance, RUNX3 has been reported to regulate the development and cytotoxic function of T cells, with its deletion leading to impaired CD8^+^ T cell activation and tumor immune escape (24). Our data extend the immune-regulatory role of RUNX3 to NK cells, which is supported by a recent study demonstrating that RUNX family members are dynamically expressed during NK cell maturation and are required for the maintenance of NK cell effector functions (44). Notably, RUNX3 induces the formation of a unique iCD8α^+^ NK cell subset by regulating CD8α expression. Compared with conventional CD8α^+^ or CD8α^-^ subsets, iCD8α^+^ NK cells exhibit superior proliferative capacity, metabolic activity, and anti-tumor cytotoxic function (45), which aligns with our observation that RUNX3 is associated with NK cell cytotoxicity and tumor cell killing.​ Given the cytotoxic role of RUNX3 in both NK cells and T cells, it is plausible that its function in NK cells is not entirely unique. However, the ability of RUNX3 to specifically drive the generation of the iCD8α^+^ NK cell subset suggests a distinct role in shaping NK cell heterogeneity and functionality that may not be fully recapitulated in T cells.

Our finding that RUNX3 mediates the crosstalk between NK cells and CD8^+^ T cells provides new insights into the coordination of anti-tumor immune responses. The synergy between NK cells and CD8^+^ T cells is essential for eliminating tumor cells. NK cells can directly kill tumor cells and secrete cytokines to promote CD8^+^ T cell activation, while activated CD8^+^ T cells further amplify the anti-tumor response through specific recognition of tumor antigens (46). Notably, Wang et al. demonstrated that high expression of RUNX3 in NK cells facilitates the interaction between NK cells and T cells, and further enhances T cell function by regulating the MHC-I, CD99, and MIF signaling pathways (47). Recent studies have indicated that transcription factors play a pivotal role in mediating such intercellular communication. For example, T-bet, a key transcription factor in both NK cells and CD8^+^ T cells, coordinates their anti-tumor functions by regulating cytokine secretion and cytotoxic molecule expression (48, 49). Our data suggest that RUNX3 may act as another critical regulator, its enrichment in NK cells and interaction with CD8^+^ T cells imply that it may participate in the reciprocal activation of these two cell populations. The hypothesis is further supported by a study demonstrating that RUNX3 promotes IFN-γ secretion in T cells (24), a critical cytokine for enhancing NK cell cytotoxicity and CD8^+^ T cell priming.

Collectively, RUNX3 exerts systematic regulatory functions within the TIME through multiple complementary mechanisms. It directly governs cytotoxic lymphocyte activity by modulating the activation, proliferation, cytotoxicity, and cytokine production of both CD8^+^ T cells and NK cells (50, 51). Beyond the direct effects on cytotoxic lymphocytes, RUNX3 also contributes to immune homeostasis by Treg function, thereby preventing excessive inflammation-driven tumorigenesis (52). Furthermore, emerging evidence suggests that RUNX3 shapes the myeloid compartment, affecting the functional status of tumor-associated macrophages (53). Together with our findings and previous reports, these multifaceted roles position RUNX3 as a key integrator of immune regulation and microenvironmental adaptation in cancer.

There are some deficiencies in the present study. First, the molecular mechanism by which RUNX3 mediates the interaction between NK cells and CD8^+^ T cells remains to be clarified, specifically, whether RUNX3 regulates the expression of co-stimulatory molecules or cytokines that are critical for intercellular crosstalk. Second, as a transcription factor, RUNX3 is presumed to function through its DNA-binding activity, while we were unable to construct DNA-binding domain mutants or transcriptional activation-deficient mutants to verify whether the observed phenotypic changes depend on its transcriptional activity. Third, although we have demonstrated the anti-tumor effects of RUNX3-regulated NK cells *in vitro*, *in vivo* studies using animal models are needed to validate the role of RUNX3 in anti-tumor immunity. Finally, clinical corroboration with larger cohorts is required to confirm the predictive value of RUNX3 in immunotherapy response.

## Conclusion

In conclusion, our study identifies a novel regulatory role of RUNX3 in NK cell-mediated anti-tumor immunity in LUAD and LIHC, providing a foundation for future exploration of RUNX3-targeted therapeutic strategies to enhance innate immune surveillance in the aggressive cancers.

## Data Availability

The original contributions presented in the study are included in the article/**Supplementary Material**. Further inquiries can be directed to the corresponding author.
