# Supplementary material for: Integrated profiling of RUNX3 in intratumoral NK cells activity through bulk and single-cell transcriptomic analysis
Source: Front Immunol. 2026 Apr 7;17:1787302. doi: 10.3389/fimmu.2026.1787302 (PMC13096070; doi:10.3389/fimmu.2026.1787302)
Supplement: Supplementary file 1 [file DataSheet1.pdf]

Supplementary materials

Integrated profiling of RUNX3 in intratumoral NK cells activity through bulk and single-cell transcriptomic analysis

Guofu Lin†, Lanlan Lin†, Jincan Zhang, Leiyuan Chen, Weitao Hu*

Supplementary Figures

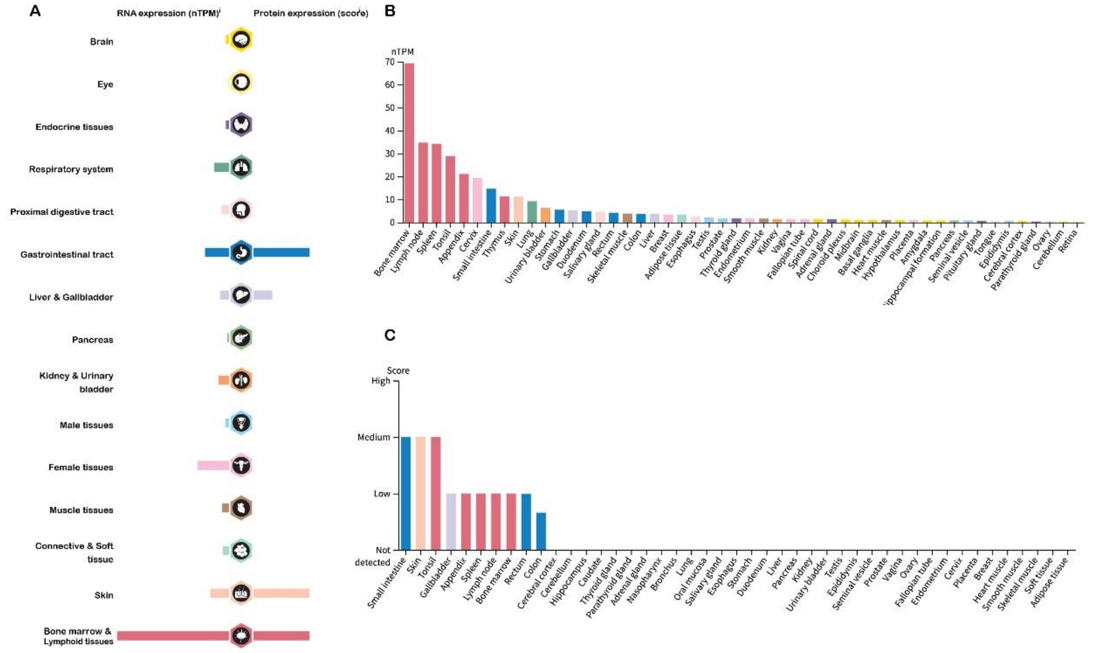

Figure S1. RUNX3 mRNA and protein expression in human organs and tissues. (A) Details of RUNX3 mRNA and protein expression in human organs and tissues. (B) RUNX3 mRNA expression in human tissues. (C) RUNX3 protein expression in human tissues.

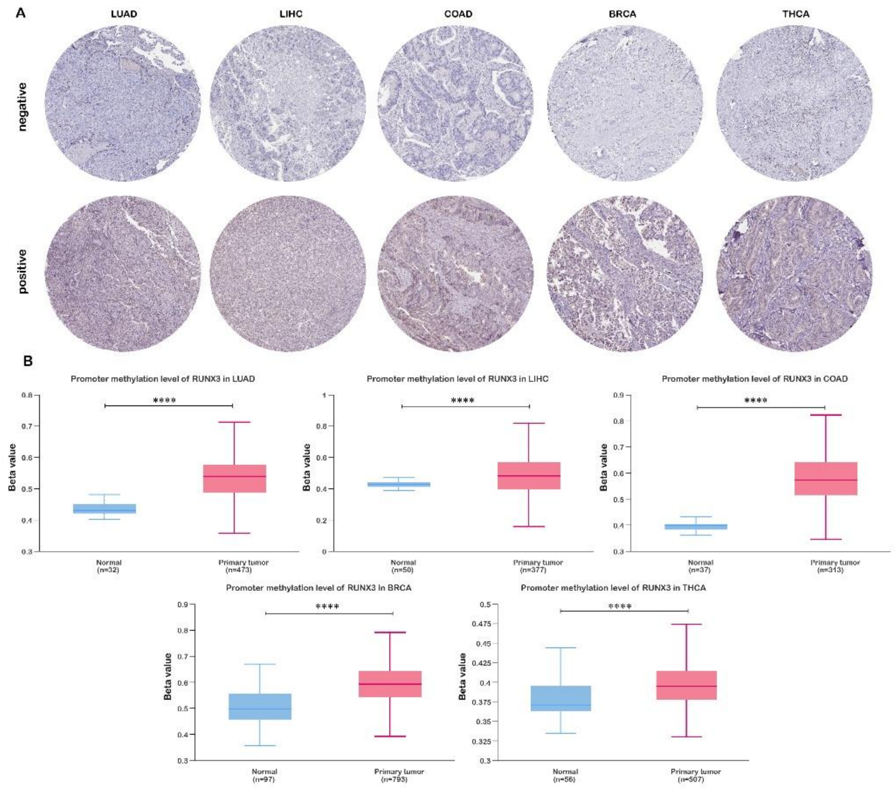

Figure S2. The protein expression and promoter methylation levels of RUNX3 in pan-cancer. (A) Representative immunohistochemical images showing the expression levels of RUNX3 in lung adenocarcinoma (LUAD), liver hepatocellular carcinoma (LIHC), colon adenocarcinoma (COAD), breast invasive carcinoma (BRCA), and thyroid carcinoma (THCA). (B) The promoter methylation levels of RUNX3 in LUAD, LIHC, COAD, BRCA, and THCA. ****$P < 0.0001$.

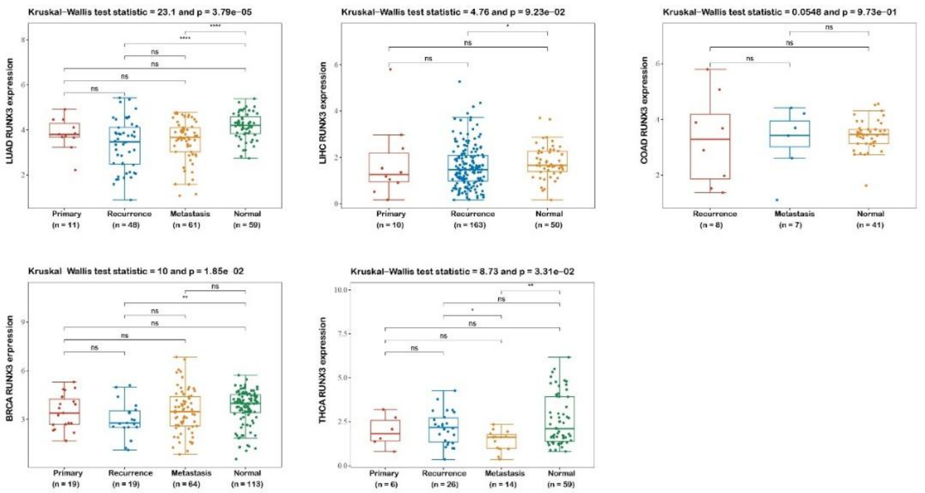

Figure S3. The expression profiles of RUNX3 across different clinical stages (primary tumor, recurrent tumor, metastatic tumor) and normal tissues in pan-cancer. *$P < 0.05$, **$P < 0.01$, ****$P < 0.0001$.

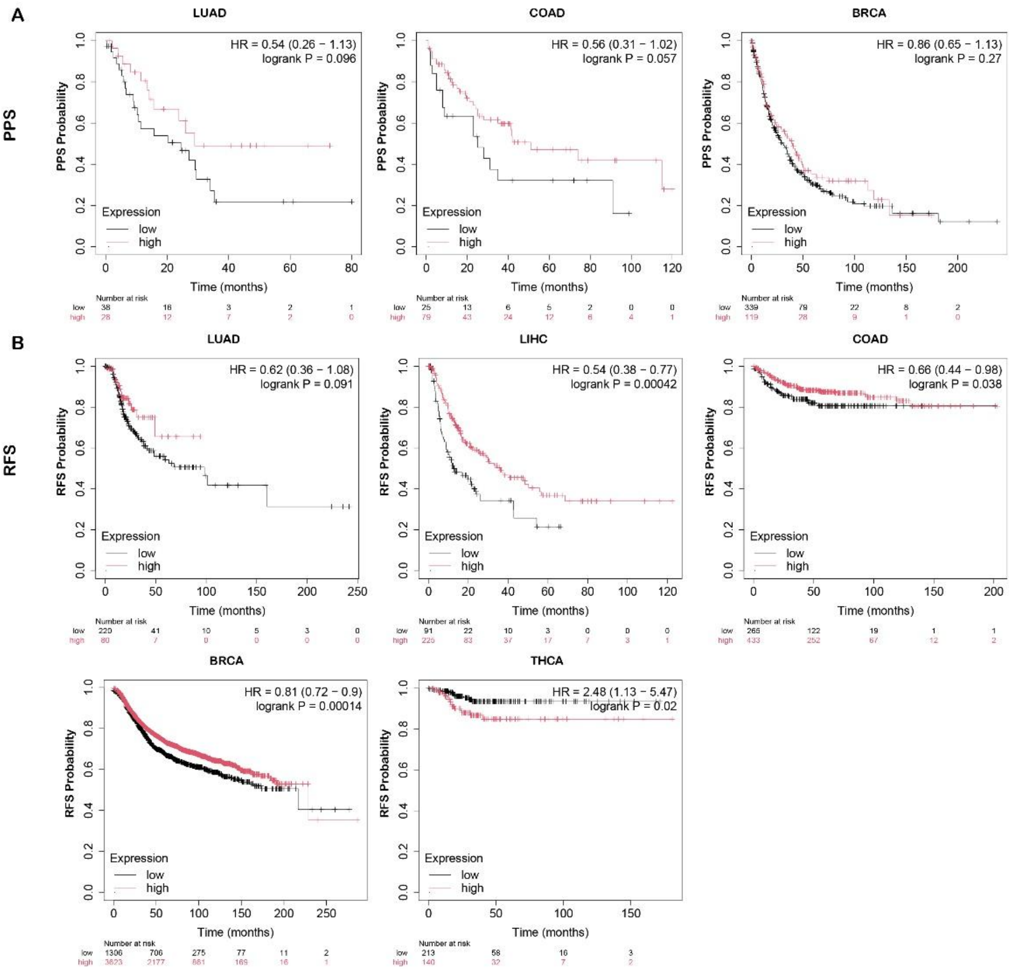

Figure S4. Significant correlation between RUNX3 expression and prognosis. (A) Association between RUNX3 expression and post-progression survival (PPS). (B) Association between RUNX3 expression and relapse-free survival (RFS).

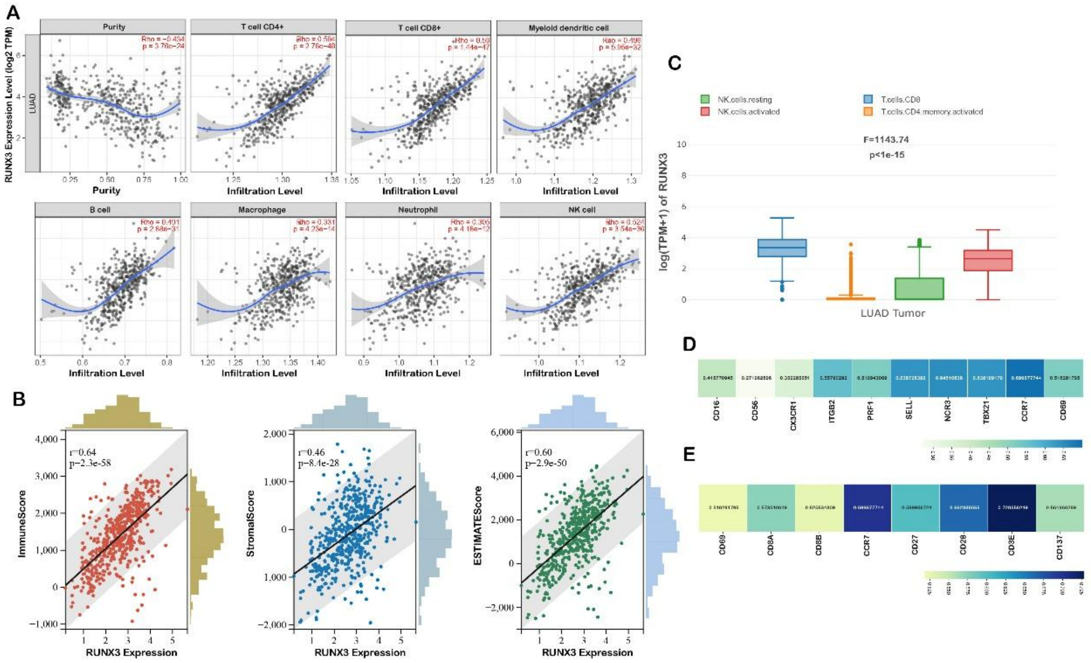

Figure S5. Correlation of RUNX3 with tumor immunity and microenvironment in LUAD tissues. (A) The association between RUNX3 expression and immune cell infiltration levels in LUAD tissues. (B) Correlations of RUNX3 expression with immune score, stromal score, and estimate score. (C) RUNX3 expression in distinct NK cell and T cell subsets. (D, E) Correlations between RUNX3 expression and marker genes of NK cell and CD8+ T cell subsets.

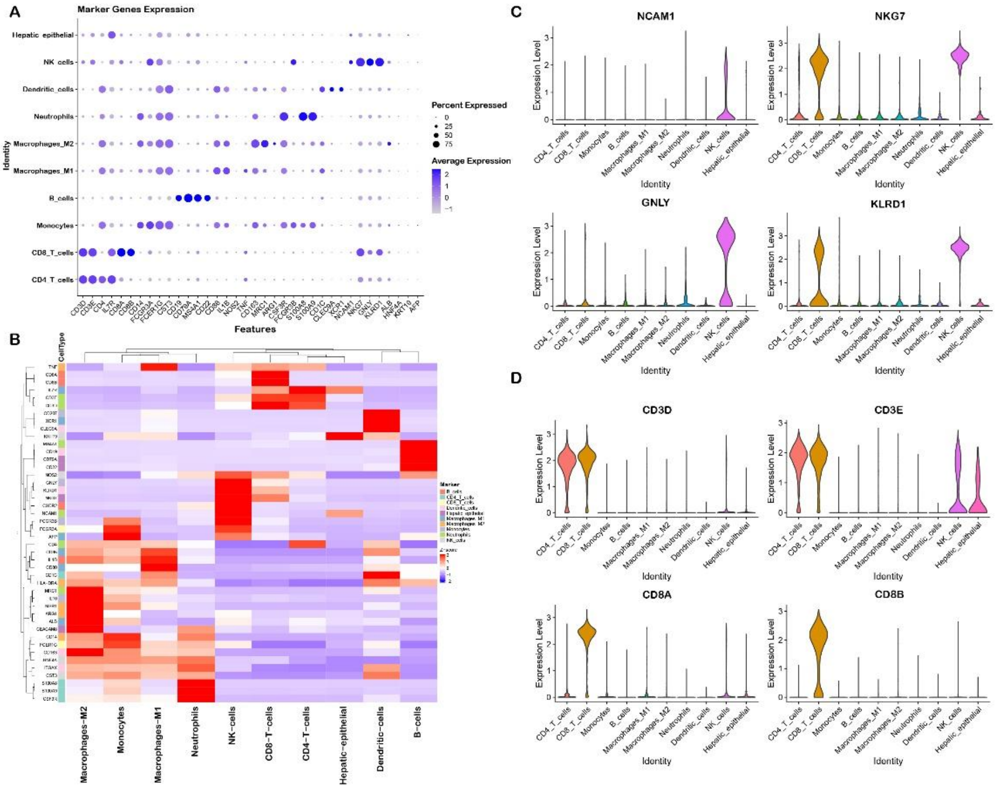

Figure S6. Characterization of immune cell subsets and the marker genes in hepatocellular carcinoma. (A, B) Dot plot and heatmap showing marker genes of immune cell subsets in the hepatocellular carcinoma single-cell dataset GSE140228. (C) Expression of marker genes in NK cell subsets. (D) Expression of marker genes in CD8+T cell subsets.

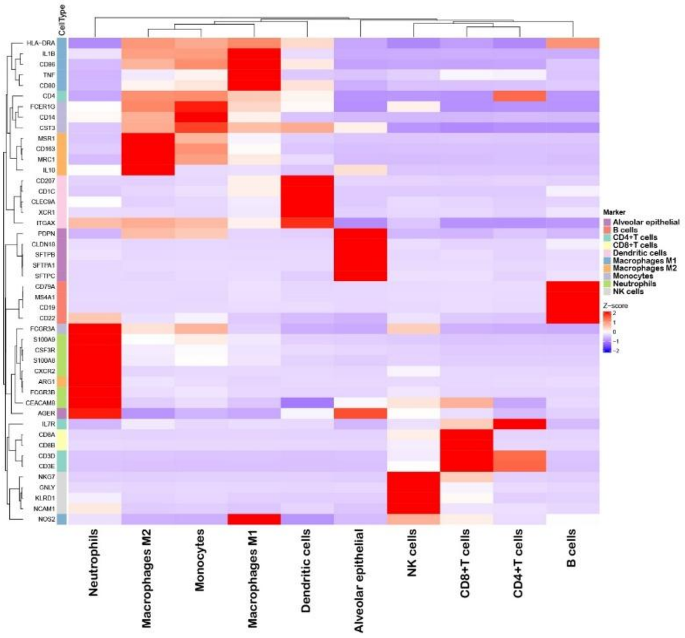

Figure S7. Heatmap illustrating the expression patterns of marker genes across distinct immune cell subsets in a single-cell RNA sequencing dataset GSE127465 of lung adenocarcinoma.

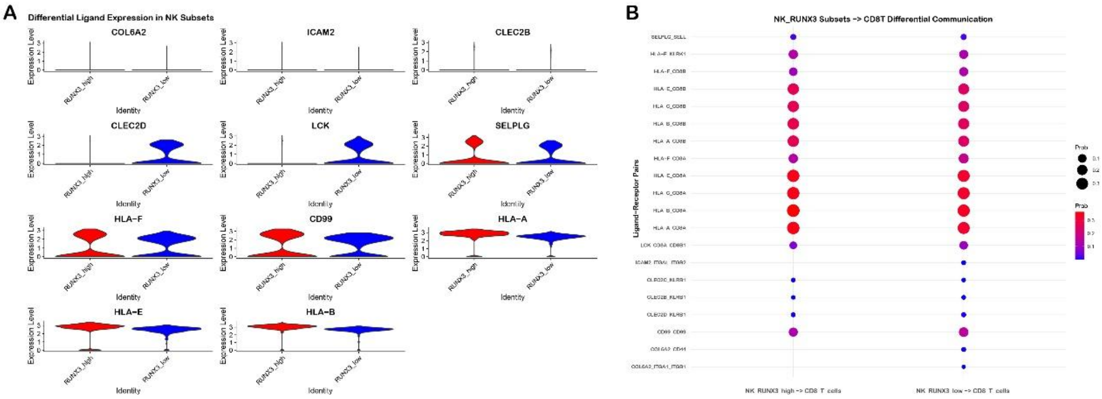

Figure S8. RUNX3 expression in NK cells reshapes receptor-ligand interactions with CD8⁺ T cells in the LUAD microenvironment. (A) Violin plots showing differential ligand expression between RUNX3-high and RUNX3-low NK subsets. (B) Dot plot illustrating receptor-ligand communication probabilities between RUNX3-high/low NK subsets and CD8⁺ T cells.

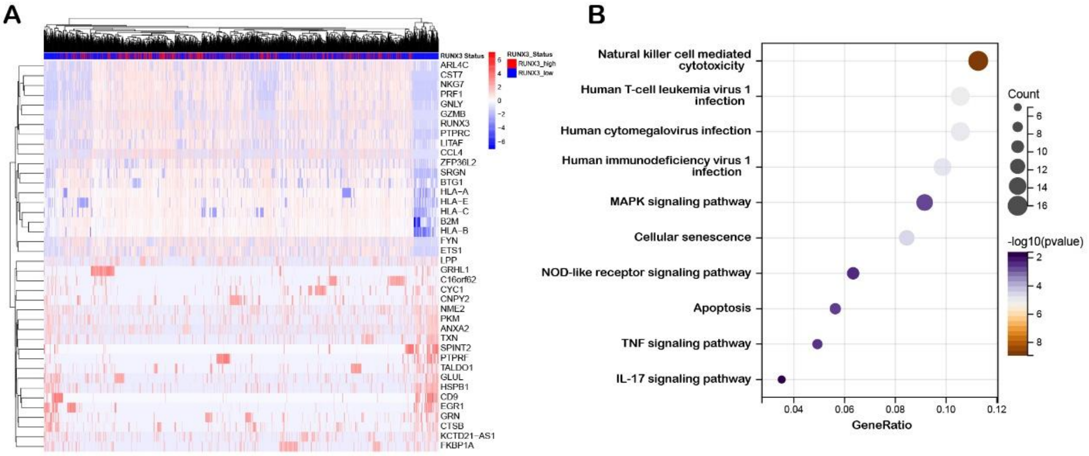

Figure S9. Differential gene expression and KEGG pathway enrichment in RUNX3^{high} and RUNX3^{low} NK cells. (A) Heatmap of differentially expressed genes (DEGs) between RUNX3^{high} and RUNX3^{low} NK cells. (B) KEGG pathway enrichment of DEGs in NK cells.

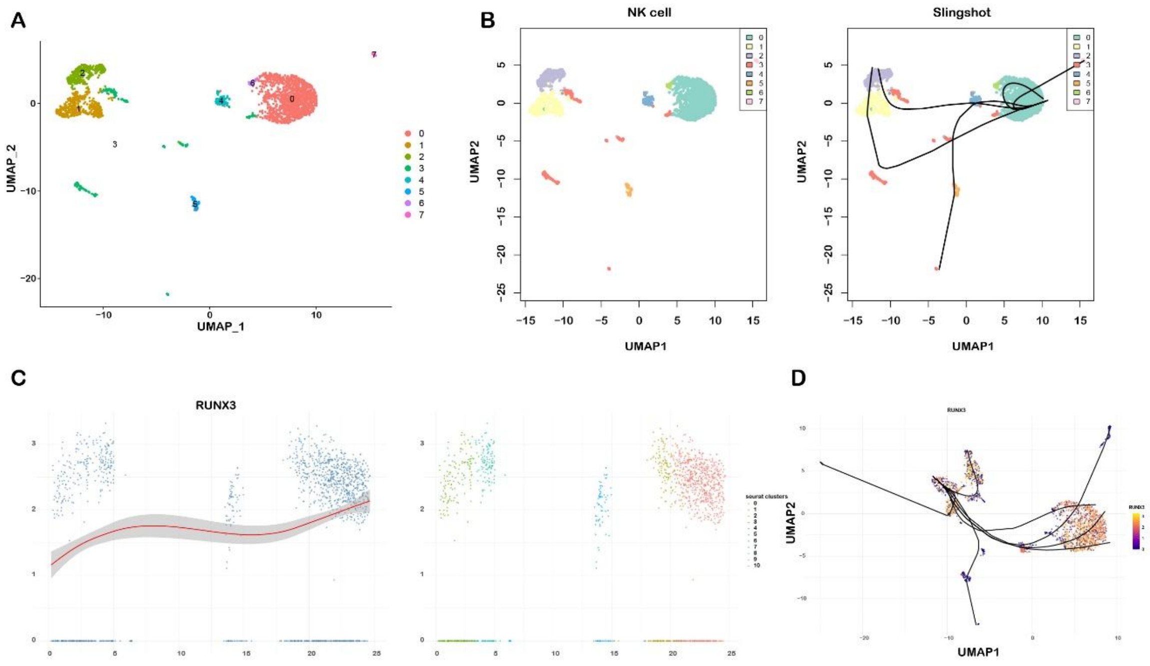

Figure S10. Pseudotime trajectory analysis of RUNX3-associated NK cell differentiation in GSE127465 dataset. (A) UMAP plot illustrating transcriptionally distinct NK cell subpopulations in LUAD microenvironment. (B) Slingshot-inferred pseudotime trajectories depicting the developmental differentiation paths of NK cells. (C) Dynamic expression profile of RUNX3 along the pseudotime axis and the spatial distribution of RUNX3 expression across NK cells. (D) Integrated pseudotime trajectory plot revealing the coupling between RUNX3 expression dynamics and the differentiation trajectories of NK cells.

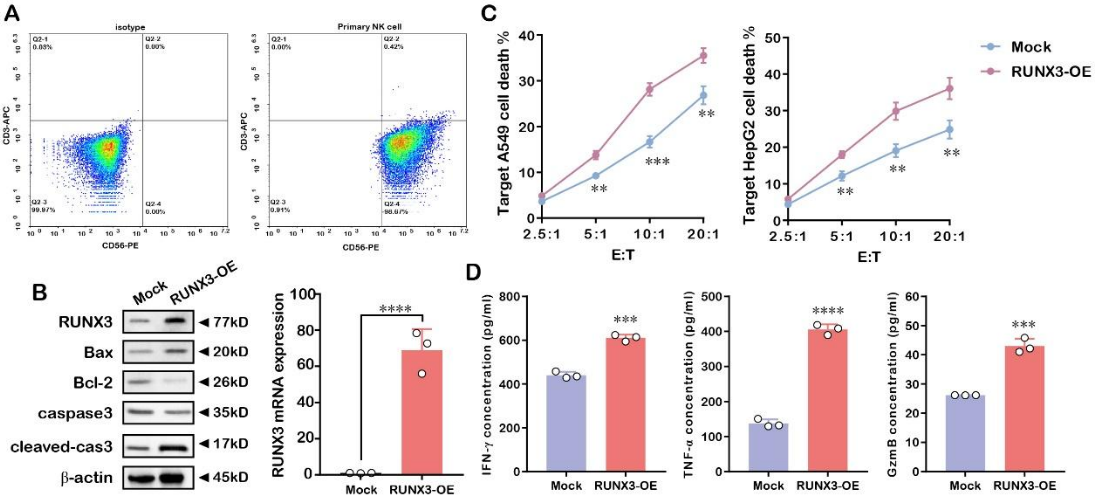

Figure S11. RUNX3 overexpression enhanced the cytotoxic function of primary NK cells. (A) Flow cytometry identification of primary human NK cells ($CD3^{-}CD56^{+}$). (B) Western blot and RT-qPCR validation of RUNX3 overexpression and its regulation of HepG2 apoptotic protein expressions. (C) Cytotoxicity assay showing enhanced cytotoxicity of RUNX3-overexpressing NK cells against A549 and HepG2 cells at indicated E:T ratios. (D) ELISA detection of elevated IFN-γ, TNF-α, and Granzyme B secretion in NK cells. $^{**}P < 0.01$, $^{***}P < 0.001$, $^{****}P < 0.0001$.
